# Supplementary material for: Quantifying the impact of clinical coding in chronic kidney disease on risk of death and COVID-19 death
Source: PLoS One. 2025 Oct 24;20(10):e0333881. doi: 10.1371/journal.pone.0333881 (PMC12551823; doi:10.1371/journal.pone.0333881)
Supplement: Supporting information 1 — Variable names and types. (DOCX) [file pone.0333881.s001.docx]

**Quantifying the impact of clinical coding in chronic kidney disease on risk of death and COVID-19 death**

**---**

**Supporting information 1**

Supplement 1: Variable names and types

| **Variable** | **Description** | **Type** |
| --- | --- | --- |
| Age | Age in years – not used in regression modelling as contained within KFRE score. Used in KFRE and not modelled as an individual predictor in regression analyses. | Numeric |
| Sex | According to recorded sex in electronic health record as male or female. Used in KFRE and not modelled as an individual predictor in regression analyses. | Categorical |
| Ethnicity | Ethnic group classification | Categorical |
| BMI group | BMI group categories | Categories |
| Socioeconomic Status (IMD deciles) | Indices of multiple deprivation (IMD) deciles | Categorical |
| eGFR | eGFR in mL/min/1.73m^2^ recalculated using the CKD-EPI 2021 equation without race modifier. Used in KFRE and not modelled as an individual predictor in regression analyses. | Numeric |
| Urine ACR | Urine ACR as recorded in the GMCR in mg/mmol. Used in KFRE and not modelled as an individual predictor in regression analyses. | Numeric |
| Diabetes | Coded diagnosis at study start | Binary |
| Hypertension | Coded diagnosis at study start | Binary |
| Coronary Heart Disease (CHD) | Coded diagnosis at study start | Binary |
| Heart Failure | Coded diagnosis at study start | Binary |
| Peripheral Arterial Disease (PAD) | Coded diagnosis at study start | Binary |
| Stroke | Coded diagnosis at study start | Binary |
| Transient Ischaemic Attack (TIA) | Coded diagnosis at study start | Binary |
| Gout | Coded diagnosis at study start | Binary |
| Myeloma | Coded diagnosis at study start | Binary |
| Non-Alcoholic Fatty Liver Disease (NAFLD) | Coded diagnosis at study start | Binary |
| Systemic Lupus Erythematosus (SLE) | Coded diagnosis at study start | Binary |
| Osteoporosis | Coded diagnosis at study start | Binary |
| Glomerulonephritis | Coded diagnosis at study start | Binary |
| Vasculitis | Coded diagnosis at study start | Binary |
| Autosomal Dominant Polycystic Kidney Disease (ADPKD) | Coded diagnosis at study start | Binary |
| Kidney Stones | Coded diagnosis at study start | Binary |
| Acute Kidney Injury (AKI) | Coded diagnosis at study start | Binary |
| Depression | Coded diagnosis at study start | Binary |
| Schizophrenia | Coded diagnosis at study start | Binary |
| Bipolar Disorder | Coded diagnosis at study start | Binary |
| Eating Disorder | Coded diagnosis at study start | Binary |
| Self-Harm and Suicidal Ideation (SI) | Coded diagnosis at study start | Binary |
| Dementia | Coded diagnosis at study start or during study | Binary |
| End of life care | Coded diagnosis of end of life care at study start | Binary |
| Coded CKD at study start | Coded diagnosis of CKD at study start | Binary |
| Coded CKD during study | Coded diagnosis of CKD during study | Binary |
| Time to death | Calculated based on number of days between study start date and date of death | Numeric |
| Time to COVID-19 death | Calculated based on number of days between study start date and date of COVID-19 death | Numeric |
| Kidney transplant | Coded diagnosis of kidney transplant at study start or during study | Binary |
| Dialysis | Coded diagnosis of dialysis at study start or during study | Binary |
