# Supplementary material for: Quantifying the impact of clinical coding in chronic kidney disease on risk of death and COVID-19 death
Source: PLoS One. 2025 Oct 24;20(10):e0333881. doi: 10.1371/journal.pone.0333881 (PMC12551823; doi:10.1371/journal.pone.0333881)
Supplement: Supporting information 2 — Clinical and demographic summary of cohort to show CKD stages 3 and 4 covariate prevalence stratified by coding status. (DOCX) [file pone.0333881.s002.docx]

**Quantifying the impact of clinical coding in chronic kidney disease on risk of death and COVID-19 death**

# ---

**Supporting information 2**

Table 1: Clinical and demographic summary of cohort to show CKD stages 3 and 4 covariate prevalence stratified by coding status

| **Variables** | **CKD Stage 3** | | **CKD Stage 4** | | **CKD Stage 5** | |
| --- | --- | --- | --- | --- | --- | --- |
|  | **Coded** | **Uncoded** | **Coded** | **Uncoded** | **Coded** | **Uncoded** |
|  | N (%) | N (%) | N (%) | N (%) | N (%) | N (%) |
| Total patients | 35307 (81.1) | 8243 (18.9) | 3512 (97.9) | 77 (2.1) | 483 (98.8) | 6 (1.2) |
| Sex | | | | | | |
| Male | 16563 (46.9) | 6669 (80.9) | 2384 (67.9) | 62 (8.1) | 335 (68.5) | |
| Age | | | | | | |
| Age group 18-39 | 224 (0.6) | 74 (0.9) | 51 (1.5) | <5 (<6) | 28 (5.7) | |
| Age group 40-59 | 2884 (8.2) | 1188 (14.4) | 321 (9.1) | 6 (7.8) | 121 (24.7) | |
| Age group 60-74 | 12261 (34.7) | 3522 (42.7) | 932 (26.5) | 16 (20.8) | 178 (36.4) | |
| Age group 75-89 | 17918 (50.7) | 3168 (38.4) | 1895 (54.0) | 36 (46.8) | 155 (31.7) | |
| Age group 90+ | 2020 (5.7) | 291 (3.5) | 313 (8.9) | 17 (22.1) | 7 (1.4) | |
| Median (IQR) years | 76 (69-83) | 72 (64-80) | 78 (70-84) | 82 (72-89) | 68 (56-79) | |
| BMI group | | | | | | |
| Low BMI <18.5 | 492 (1.4) | 77 (0.9) | 53 (1.5) | 5 (6.5) | 7 (1.4) | |
| Normal BMI 18.5-24.9 | 8041 (22.8) | 1797 (21.8) | 841 (23.9) | 27 (35.1) | 128 (26.2) | |
| Overweight BMI 25-29.9 | 13389 (37.9) | 3357 (40.7) | 1305 (37.2) | 23 (29.9) | 169 (34.6) | |
| Obese BMI 30-39.9 | 11419 (32.3) | 2584 (31.3) | 1074 (30.6) | 16 (20.8) | 140 (28.6) | |
| Severely obese BMI >=40 | 1529 (4.3) | 265 (3.2) | 179 (5.1) | <5 (<6) | 28 (5.7) | |
| Missing | 437 (1.2) | 163 (2.0) | 60 (1.7) | <5 (<6) | 17 (3.5) | |
| Ethnicity | | | | | | |
| White or White British | 28061 (79.5) | 6257 (75.9) | 2587 (73.7) | 48 (62.3) | 302 (61.8) | |
| Asian or Asian British | 1990 (5.6) | 523 (6.3) | 309 (8.8) | <5 (<6) | 84 (17.2) | |
| Black or Black British | 845 (2.4) | 324 (3.9) | 95 (2.7) | <5 (<6) | 34 (7.0) | |
| Mixed | 205 (0.6) | 75 (0.9) | 19 (0.5) | <5 (<6) | 6 (1.2) | |
| Other ethnic groups | 3485 (9.9) | 885 (10.7) | 422 (12.0) | 16 (20.8) | 52 (10.6) | |
| Missing | 721 (2.0) | 179 (2.2) | 80 (2.3) | 11 (14.3) | 11 (2.2) | |
| IMD deciles | | | | | | |
| 1 (most deprived) | 7090 (20.1) | 1734 (21.0) | 810 (23.1) | 13 (16.9) | 158 (32.3) | |
| 2 | 5060 (14.3) | 1019 (12.4) | 502 (14.3) | 14 (18.2) | 73 (14.9) | |
| 3 | 4072 (11.5) | 923 (11.2) | 359 (10.2) | 9 (11.7) | 65 (13.3) | |
| 4 | 2866 (8.1) | 638 (7.7) | 298 (8.5) | 9 (11.7) | 30 (6.1) | |
| 5 | 2904 (8.2) | 608 (7.4) | 258 (7.3) | <5 (<6) | 26 (5.3) | |
| 6 | 2206 (6.2) | 468 (5.7) | 193 (5.5) | 6 (7.8) | 36 (7.4) | |
| 7 | 2889 (8.2) | 664 (8.1) | 303 (8.6) | 6 (7.8) | 31 (6.3) | |
| 8 | 3341 (9.5) | 845 (10.3) | 337 (9.6) | 9 (11.7) | 26 (5.3) | |
| 9 | 2729 (7.7) | 773 (9.4) | 274 (7.8) | <5 (<6.0) | 26 (5.3) | |
| 10 (least deprived) | 2143 (6.1) | 571 (6.9) | 177 (5.0) | <5 (<6.0) | 18 (3.7) | |
| Missing | 7 (<0.1) | <5 (<0.1) | <5 (<0.1) | <5 (<6.0) | <5 (<1.0) | |
| Diagnoses at study start | | | | | | |
| Diabetes | 10915 (30.9) | 2313 (28.1) | 1662 (47.3) | 20 (26.0) | 216 (44.2) | |
| Hypertension | 25440 (72.1) | 4923 (59.7) | 2858 (81.4) | 43 (55.8) | 410 (83.8) | |
| SLE | 116 (0.3) | 14 (0.2) | 14 (0.4) | <5 (<6.0) | <5 (<1.0) | |
| Gout | 4902 (13.9) | 964 (11.7) | 901 (25.7) | 9 (11.7) | 119 (24.3) | |
| NAFLD | 539 (1.5) | 127 (1.5) | 52 (1.5) | <5 (<6.0) | 6 (1.2) | |
| Myeloma | 80 (0.2) | 15 (0.2) | 17 (0.5) | <5 (<6.0) | <5 (<1.0) | |
| Osteoporosis | 3224 (9.1) | 363 (4.4) | 246 (7.0) | <5 (<6.0) | 20 (4.1) | |
| CHD | 7896 (22.4) | 1774 (21.5) | 1049 (29.9) | 21 (27.3) | 110 (22.5) | |
| Heart failure | 3052 (8.6) | 632 (7.7) | 565 (16.1) | 13 (16.9) | 64 (13.1) | |
| PAD | 1729 (4.9) | 323 (3.9) | 285 (8.1) | 6 (7.8) | 39 (8.0) | |
| Stroke | 2750 (7.8) | 535 (6.5) | 347 (9.9) | 6 (7.8) | 41 (8.4) | |
| TIA | 2048 (5.8) | 408 (4.9) | 237 (6.7) | 6 (7.8) | 24 (4.9) | |
| ADPKD | 586 (1.7) | 19 (0.2) | 246 (7.0) | <5 (<6.0) | 95 (19.4) | |
| Glomerulonephritis | 163 (0.5) | 14 (0.2) | 91 (2.6) | <5 (<6.0) | 28 (5.7) | |
| Kidney stones | 844 (2.4) | 191 (2.3) | 125 (3.6) | <5 (<6.0) | 16 (3.3) | |
| Vasculitis | 252 (0.7) | 43 (0.5) | 41 (1.2) | <5 (<6.0) | 6 (1.2) | |
| Acute kidney injury | 6388 (18.1) | 841 (10.2) | 808 (23.0) | 16 (20.8) | 149 (30.5) | |
| Depression | 11127 (31.5) | 2096 (25.4) | 916 (26.1) | 16 (20.8) | 144 (29.4) | |
| Schizophrenia | 667 (1.9) | 184 (2.2) | 70 (2.0) | <5 (<6.0) | <5 (<1.0) | |
| Bipolar disorder | 303 (0.9) | 67 (0.8) | 33 (0.9) | <5 (<6.0) | <5 (<1.0) | |
| Eating disorder | 580 (1.6) | 104 (1.3) | 68 (1.9) | <5 (<6.0) | 16 (3.3) | |
| Self-harm and suicidal ideation | 1044 (3.0) | 223 (2.7) | 85 (2.4) | <5 (<6.0) | 16 (3.3) | |
| COVID-19 vaccination | | | | | | |
| Vaccinated | 33648 (95.3) | 7706 (93.5) | 3265 (93.0) | 57 (74.0) | 435 (89.0) | |
| Measurements | | | | | | |
| eGFR (median(IQR)) | 46 (39-52) | 51 (47-55) | 25 (22-28) | 26 (23-28) | 11 (8-13) | |
| uACR (median(IQR)) | 1.8 (0.8-5.2) | 1.4 (0.7-3.7) | 6.3 (1.9-23.6) | 5.4 (1.8-208.0) | 39.3 (9.0-97.7) | |
| **Key:** BMI = Body mass index; SLE = systemic lupus erythematosus; NAFLD = non-alcoholic fatty liver disease; CHD = coronary heart disease; PAD = peripheral arterial disease; TIA = transient ischaemic attack; ADPKD = autosomal dominant polycystic kidney disease; eGFR = estimated glomerular filtration rate; uACR = urine albumin creatinine ratio. N and % are aggregated for both coded and uncoded CKD stage 5. | | | | | | |
