# Supplementary material for: Quantifying the impact of clinical coding in chronic kidney disease on risk of death and COVID-19 death
Source: PLoS One. 2025 Oct 24;20(10):e0333881. doi: 10.1371/journal.pone.0333881 (PMC12551823; doi:10.1371/journal.pone.0333881)
Supplement: Supporting information 3 — (DOCX) [file pone.0333881.s003.docx]

**Quantifying the impact of clinical coding in chronic kidney disease on risk of death and COVID-19 death**

**---**

**Supporting information 3**

***Propensity Score Matching***

- Propensity score matching is used to balance covariates a range of covariates between patients with coded and uncoded CKD stages 3 and 4.
- All models measured the impact of the following predictors: coded CKD, sex, age group, ethnicity, BMI group, IMD, and the following conditions at study start – diabetes, hypertension, heart failure, CHD, PAD, stroke, TIA, osteoporosis, myeloma, NAFLD, gout, SLE, vasculitis, glomerulonephritis, ADPKD, kidney stones, AKI, depression, schizophrenia, bipolar disorder, eating disorder, and self-harm and suicidal ideation. Variables not appearing in the models were not statistically significant. Non-significant predictors in the model were retained based on model goodness-of-fit tests.
- Primary analyses describe a conditional hazard of death, whereas sensitivity analyses using competing risk regression describe a marginal hazard of death.
- Mean absolute standardised mean difference (SMD) is the average total SMD score. Thresholds for overall balance:

| **Excellent balance** | *<0.1* |
| --- | --- |
| **Moderate balance** | *0.1-0.2* |
| **Poor balance** | *>0.2* |

***CKD Stage 3: Risk of Death – Propensity Score Matching***

- Moderate balance exists before matching (table 1). PSM 1:1 and 1:5 with replacement both offer excellent balance of covariates between coded and uncoded CKD stage 3.

*Table 1: Summary of covariates balance between coded and uncoded CKD stage 3, prior to matching and after matching using PSM 1:1, and 1:5 matching with replacement.*

|  | **Before Matching** | | | **After Matching 1:1** | | | **After Matching 1:5 w/ replacement** | | |
| --- | --- | --- | --- | --- | --- | --- | --- | --- | --- |
|  | **Uncoded** | **Coded** | **SMD** | **Uncoded** | **Coded** | **SMD** | **Uncoded** | **Coded** | **SMD** |
| n | 8080 | 34863 |  | 8080 | 8080 |  | 8080 | 40400 |  |
| **Sex = M (%)** | 6563 (81.2) | 16375 (47.0) | 0.764 | 6563 (81.2) | 6526 (80.8) | **0.012** | 6563 (81.2) | 32618 (80.7) | **0.012** |
| **Age group (%)** |  |  | 0.319 |  |  | **0.028** |  |  | **0.026** |
| 18-39 | 63 (0.8) | 212 (0.6) |  | 63 (0.8) | 49 (0.6) |  | 63 (0.8) | 252 (0.6) |  |
| 40-59 | 1162 (14.4) | 2831 (8.1) |  | 1162 (14.4) | 1112 (13.8) |  | 1162 (14.4) | 5591 (13.8) |  |
| 60-74 | 3484 (43.1) | 12138 (34.8) |  | 3484 (43.1) | 3512 (43.5) |  | 3484 (43.1) | 17518 (43.4) |  |
| 75-89 | 3101 (38.4) | 17722 (50.8) |  | 3101 (38.4) | 3131 (38.8) |  | 3101 (38.4) | 15715 (38.9) |  |
| 90+ | 270 (3.3) | 1960 (5.6) |  | 270 (3.3) | 276 (3.4) |  | 270 (3.3) | 1324 (3.3) |  |
| **BMI group** |  |  | **0.089** |  |  | **0.015** |  |  | **0.024** |
| <18.5 | 77 (1.0) | 492 (1.4) |  | 1797 (22.2) | 1778 (22.0) |  | 77 (1.0) | 390 (1.0) |  |
| 18.5-24.9 | 1797 (22.2) | 8038 (23.1) |  | 77 (1.0) | 70 (0.9) |  | 1797 (22.2) | 8805 (21.8) |  |
| 25-29.9 | 3357 (41.5) | 13388 (38.4) |  | 265 (3.3) | 259 (3.2) |  | 3357 (41.5) | 17173 (42.5) |  |
| 30-39.9 | 2584 (32.0) | 11417 (32.7) |  | 3357 (41.5) | 3405 (42.1) |  | 2584 (32.0) | 12821 (31.7) |  |
| >=40 | 265 (3.3) | 1528 (4.4) |  | 2584 (32.0) | 2568 (31.8) |  | 265 (3.3) | 1211 (3.0) |  |
| **IMD decile** |  |  | 0.101 |  |  | 0.559 |  |  | **0.038** |
| 1 | 1703 (21.1) | 7005 (20.1) |  | 1703 (21.1) | 1623 (20.1) |  | 1703 (21.1) | 8201 (20.3) |  |
| 2 | 996 (12.3) | 4991 (14.3) |  | 996 (12.3) | 1044 (12.9) |  | 996 (12.3) | 5169 (12.8) |  |
| 3 | 905 (11.2) | 4032 (11.6) |  | 905 (11.2) | 915 (11.3) |  | 905 (11.2) | 4493 (11.1) |  |
| 4 | 624 (7.7) | 2836 (8.1) |  | 624 (7.7) | 613 (7.6) |  | 624 (7.7) | 3162 (7.8) |  |
| 5 | 600 (7.4) | 2880 (8.3) |  | 600 (7.4) | 651 (8.1) |  | 600 (7.4) | 3192 (7.9) |  |
| 6 | 461 (5.7) | 2187 (6.3) |  | 461 (5.7) | 486 (6.0) |  | 461 (5.7) | 2481 (6.1) |  |
| 7 | 652 (8.1) | 2851 (8.2) |  | 652 (8.1) | 670 (8.3) |  | 652 (8.1) | 3307 (8.2) |  |
| 8 | 833 (10.3) | 3307 (9.5) |  | 833 (10.3) | 835 (10.3) |  | 833 (10.3) | 4119 (10.2) |  |
| 9 | 756 (9.4) | 2682 (7.7) |  | 756 (9.4) | 719 (8.9) |  | 756 (9.4) | 3678 (9.1) |  |
| 10 | 550 (6.8) | 2092 (6.0) |  | 550 (6.8) | 524 (6.5) |  | 550 (6.8) | 2598 (6.4) |  |
| **Ethnicity** |  |  | 0.11 |  |  | **0.027** |  |  | **0.041** |
| White | 6130 (75.9) | 27693 (79.4) |  | 6130 (75.9) | 6174 (76.4) |  | 6130 (75.9) | 31284 (77.4) |  |
| Asian or Asian British | 517 (6.4) | 1974 (5.7) |  | 517 (6.4) | 507 (6.3) |  | 517 (6.4) | 2497 (6.2) |  |
| Black or Black British | 318 (3.9) | 832 (2.4) |  | 318 (3.9) | 289 (3.6) |  | 318 (3.9) | 1528 (3.8) |  |
| Mixed | 69 (0.9) | 201 (0.6) |  | 69 (0.9) | 59 (0.7) |  | 69 (0.9) | 284 (0.7) |  |
| Other Ethnic Groups | 872 (10.8) | 3458 (9.9) |  | 872 (10.8) | 889 (11.0) |  | 872 (10.8) | 3991 (9.9) |  |
| Missing | 174 (2.2) | 705 (2.0) |  | 174 (2.2) | 162 (2.0) |  | 174 (2.2) | 816 (2.0) |  |
| **Diagnoses** |  |  |  |  |  |  |  |  |  |
| Diabetes | 2304 (28.5) | 10878 (31.2) | **0.059** | 2304 (28.5) | 2244 (27.8) | **0.017** | 2304 (28.5) | 10942 (27.1) | **0.032** |
| Hypertension | 4853 (60.1) | 25199 (72.3) | 0.26 | 4853 (60.1) | 4879 (60.4) | **0.007** | 4853 (60.1) | 24501 (60.6) | **0.012** |
| SLE | 14 (0.2) | 116 (0.3) | **0.032** | 14 (0.2) | 14 (0.2) | **0.001** | 14 (0.2) | 71 (0.2) | **0.001** |
| Gout | 953 (11.8) | 4863 (13.9) | **0.064** | 953 (11.8) | 953 (11.8) | **0.010** | 953 (11.8) | 4629 (11.5) | **0.011** |
| NAFLD | 127 (1.6) | 539 (1.5) | **0.002** | 127 (1.6) | 114 (1.4) | **0.013** | 127 (1.6) | 495 (1.2) | **0.03** |
| Myeloma | 15 (0.2) | 80 (0.2) | **0.01** | 15 (0.2) | 12 (0.1) | **0.009** | 15 (0.2) | 61 (0.2) | **0.008** |
| Osteoporosis | 352 (4.4) | 3190 (9.2) | 0.192 | 352 (4.4) | 341 (4.2) | **0.007** | 352 (4.4) | 1671 (4.1) | **0.011** |
| CHD | 1746 (21.6) | 7839 (22.5) | **0.021** | 1746 (21.6) | 1715 (21.2) | **0.009** | 1746 (21.6) | 8162 (20.2) | **0.035** |
| Heart failure | 619 (7.7) | 3020 (8.7) | **0.037** | 619 (7.7) | 550 (6.8) | **0.033** | 619 (7.7) | 2552 (6.3) | **0.053** |
| PAD | 317 (3.9) | 1719 (4.9) | **0.049** | 317 (3.9) | 298 (3.7) | **0.012** | 317 (3.9) | 1377 (3.4) | **0.027** |
| Stroke | 521 (6.4) | 2716 (7.8) | **0.052** | 521 (6.4) | 461 (5.7) | **0.031** | 521 (6.4) | 2189 (5.4) | **0.044** |
| TIA | 401 (5.0) | 2033 (5.8) | **0.038** | 401 (5.0) | 370 (4.6) | **0.018** | 401 (5.0) | 1712 (4.2) | **0.035** |
| ADPKD | 19 (0.2) | 569 (1.6) | 0.146 | 19 (0.2) | 13 (0.2) | 0.17 | 19 (0.2) | 94 (0.2) | **0.001** |
| Glomerulonephritis | 14 (0.2) | 159 (0.5) | **0.051** | 14 (0.2) | 15 (0.2) | **0.003** | 14 (0.2) | 58 (0.1) | **0.007** |
| Kidney stones | 189 (2.3) | 834 (2.4) | **0.003** | 189 (2.3) | 153 (1.9) | **0.031** | 189 (2.3) | 735 (1.8) | **0.036** |
| Vasculitis | 43 (0.5) | 250 (0.7) | **0.023** | 43 (0.5) | 42 (0.5) | **0.002** | 43 (0.5) | 183 (0.5) | **0.011** |
| Acute kidney injury | 828 (10.2) | 6324 (18.1) | 0.228 | 828 (10.2) | 745 (9.2) | **0.035** | 828 (10.2) | 3700 (9.2) | **0.037** |
| Depression | 2067 (25.6) | 11005 (31.6) | 0.133 | 2067 (25.6) | 2003 (24.8) | **0.018** | 2067 (25.6) | 9663 (23.9) | **0.039** |
| Schizophrenia | 182 (2.3) | 664 (1.9) | **0.024** | 182 (2.3) | 148 (1.8) | **0.030** | 182 (2.3) | 673 (1.7) | **0.042** |
| Bipolar disorder | 67 (0.8) | 303 (0.9) | **0.004** | 67 (0.8) | 57 (0.7) | **0.014** | 67 (0.8) | 281 (0.7) | **0.015** |
| Eating disorder | 103 (1.3) | 577 (1.7) | **0.032** | 103 (1.3) | 85 (1.1) | **0.021** | 103 (1.3) | 429 (1.1) | **0.02** |
| Self-harm and suicidal ideation | 221 (2.7) | 1037 (3.0) | **0.014** | 221 (2.7) | 199 (2.5) | 0.299 | 221 (2.7) | 914 (2.3) | **0.03** |
| Mean absolute SMD |  |  | 0.105 |  |  | 0.050 |  |  | 0.030 |
| **Key:** SMD = standardized mean difference. Excellent balance of covariates indicated by SMD value in bold i.e. <0.10. Mean absolute SMD values colour coded according to thresholds: red = poor balance; orange = moderate balance; green = excellent balance. | | | | | | | | | |

***CKD Stage 3: Risk of Death – PSM***

- In both models using PSM (table 2), coded CKD stage 3 (versus uncoded CKD stage 3) was associated with a consistently significantly lower hazard of death. Specifically, in the PSM 1:5 model, coded CKD stage 3 was associated with 20% lower hazard of death (HR 0.80, CIs 0.75-0.84, p=<0.0001); mortality risk increases significantly with age; male sex was associated with a 14% higher hazard of death (HR 1.14, CIs 1.07-1.20, p=<0.0001; low BMI (<18.5) was associated with increased hazard of death (HR 1.98, CIs 1.73-2.28, p=<0.0001) whereas overweight/obese BMI was associated with ~30% lower hazard of death; a one decile increase in IMD was associated with a 6% lower hazard of death (HR 0.94, CIs 0.93-0.95, p=<0.0001); of physical health conditions - heart failure (HR 1.83, CIs 1.71-1.95, p=<0.0001), PAD (HR 1.64, CIs 1.50-1.79, p=<0.0001), stroke (HR 1.44, CIs 1.34-1.55, p=<0.0001, and diabetes (HR 1.44, CIs 1.37-1.51, p=<0.0001) were all associated with increased hazard of death; and of mental health conditions – depression (HR 1.14, CIs 1.08-1.20, p=<0.0001), schizophrenia (HR 1.57, CIs 1.35-1.82, p=<0.0001), eating disorder (HR 1.37, CIs 1.16-1.61, p=0.0002) and self-harm and suicidal ideation (HR 1.18, CIs 1.01-1.39, p=0.04) were all associated with increased hazard of death.

*Table 2: Cox PH models assessing risk of death in patients with coded and uncoded CKD stage 3 using PSM 1:1 and PSM 1:5 with replacement*

|  | **CKD Stage 3 – Risk of Death** | | | | | |
| --- | --- | --- | --- | --- | --- | --- |
|  | **PSM 1:1** | | | **PSM 1:5 w/ replacement** | | |
| **Variable** | **HR** | **P-value** | **95% CIs** | **HR** | **P-value** | **95% CIs** |
| **Coded CKD** | 0.79 | **<0.0001** | 0.74-0.85 | 0.80 | **<0.0001** | 0.75-0.84 |
| **Male Sex** | 1.01 | 0.83 | 0.92-1.11 | 1.14 | **<0.0001** | 1.07-1.20 |
| **Age group 18-39** | 0.20 | 0.03 | 0.05-0.80 | 0.11 | **0.0002** | 0.04-0.36 |
| **Age group 40-59** | 0.43 | **<0.0001** | 0.34-0.54 | 0.43 | **<0.0001** | 0.38-0.50 |
| **Age group 75-89** | 2.92 | **<0.0001** | 2.66-3.20 | 2.92 | **<0.0001** | 2.76-3.08 |
| **Age group 90+** | 8.25 | **<0.0001** | 7.18-9.48 | 9.21 | **<0.0001** | 8.48-10.00 |
| **BMI <18.5** | 1.93 | **<0.0001** | 1.54-2.44 | 1.98 | **<0.0001** | 1.73-2.28 |
| **BMI 25-29.9** | 0.70 | **<0.0001** | 1.54-2.44 | 0.70 | **<0.0001** | 0.66-0.74 |
| **BMI 30-39.9** | 0.72 | **<0.0001** | 0.65-0.80 | 0.72 | **<0.0001** | 0.68-0.77 |
| **BMI >=40** | 1.02 | **0.84** | 0.83-1.27 | 1.01 | 0.94 | 0.88-1.15 |
| **IMD** | 0.95 | **<0.0001** | 0.93-0.96 | 0.94 | **<0.0001** | 0.93-0.95 |
| **Diabetes** | 1.45 | **<0.0001** | 1.33-1.57 | 1.44 | **<0.0001** | 1.37-1.51 |
| **Hypertension** | 1.14 | **0.001** | 1.05-1.24 | 1.14 | **<0.0001** | 1.09-1.20 |
| **Gout** | - | - | - | - | - | - |
| **Myeloma** | 2.88 | **0.002** | 1.49-5.57 | - | - | - |
| **Osteoporosis** | 1.18 | **0.02** | 1.02-1.36 | 1.24 | **<0.0001** | 1.14-1.35 |
| **CAD** | 1.20 | **<0.0001** | 1.11-1.31 | 1.21 | **<0.0001** | 1.15-1.28 |
| **HF** | 1.71 | **<0.0001** | 1.53-1.90 | 1.83 | **<0.0001** | 1.71-1.95 |
| **PAD** | 1.50 | **<0.0001** | 1.30-1.74 | 1.64 | **<0.0001** | 1.50-1.79 |
| **Stroke** | 1.38 | **<0.0001** | 1.22-1.55 | 1.44 | **<0.0001** | 1.34-1.55 |
| **TIA** | - | - | - | - | - | - |
| **AKI** | - | - | - | 1.09 | **0.02** | 1.01-1.16 |
| **Depression** | 1.13 | **0.004** | 1.04-1.24 | 1.14 | **<0.0001** | 1.08-1.20 |
| **Schizophrenia** | 1.43 | **0.004** | 1.12-1.81 | 1.57 | **<0.0001** | 1.35-1.82 |
| **Eating disorder** | 1.45 | **0.004** | 1.12-1.88 | 1.37 | **0.0002** | 1.16-1.61 |
| **Self-harm and SI** | - | - | - | 1.18 | **0.04** | 1.01-1.39 |
| **Reference categories** | Coding status: uncoded at study start; Sex: Female; Age group: 40-59; BMI: 18.5-24.9 (healthy weight); Comorbidities: absence of diagnosis at study starts | | | | | |
| **Statistically significant** | P-values in bold | | | | | |
| **Not statistically significant** | Cells including - | | | | | |

***CKD Stage 3: Risk of COVID-19 Death – Propensity Score Matching***

- There is excellent balance for individual diagnoses with moderate balance overall before matching (table 3). PSM 1:1 and 1:5 with replacement both offer excellent balance of covariates between coded and uncoded CKD stage 3.

*Table 3: PSM for patients with CKD stage 3 using 1:1 matching, and 1:5 matching with replacement.*

|  | **Before Matching** | | | **After Matching 1:1** | | | **After Matching 1:5 w/ replacement** | | |
| --- | --- | --- | --- | --- | --- | --- | --- | --- | --- |
|  | **Uncoded** | **Coded** | **SMD** | **Uncoded** | **Coded** | **SMD** | **Uncoded** | **Coded** | **SMD** |
| n | 8001 | 34636 |  | 8001 | 8001 |  | 8001 | 40005 |  |
| **Sex = M (%)** | 6515 (81.4) | 16274 (47.0) | 0.77 | 6515 (81.4) | 6485 (81.1) | **0.01** | 6515 (81.4) | 32350 (80.9) | **0.014** |
| **Age group (%)** |  |  | 0.325 |  |  | **0.033** |  |  | **0.03** |
| 18-39 | 63 (0.8) | 212 (0.6) |  | 63 (0.8) | 47 (0.6) |  | 63 (0.8) | 233 (0.6) |  |
| 40-59 | 1162 (14.5) | 2828 (8.2) |  | 1162 (14.5) | 1112 (13.9) |  | 1162 (14.5) | 5656 (14.1) |  |
| 60-74 | 3472 (43.4) | 12088 (34.9) |  | 3472 (43.4) | 3494 (43.7) |  | 3472 (43.4) | 17355 (43.4) |  |
| 75-89 | 3044 (38.0) | 17607 (50.8) |  | 3044 (38.0) | 3099 (38.7) |  | 3044 (38.0) | 15500 (38.7) |  |
| 90+ | 260 (3.2) | 1901 (5.5) |  | 260 (3.2) | 249 (3.1) |  | 260 (3.2) | 1261 (3.2) |  |
| **BMI group** |  |  |  |  |  | **0.032** |  |  | **0.053** |
| <18.5 | 68 (0.8) | 472 (1.4) | **0.094** | 68 (0.8) | 66 (0.8) |  | 68 (0.8) | 304 (0.8) |  |
| 18.5-24.9 | 1770 (22.1) | 7960 (23.0) |  | 1770 (22.1) | 1738 (21.7) |  | 1770 (22.1) | 8497 (21.2) |  |
| 25-29.9 | 3331 (41.6) | 13311 (38.4) |  | 3331 (41.6) | 3437 (43.0) |  | 3331 (41.6) | 17465 (43.7) |  |
| 30-39.9 | 2572 (32.1) | 11373 (32.8) |  | 2572 (32.1) | 2529 (31.6) |  | 2572 (32.1) | 12686 (31.7) |  |
| >=40 | 260 (3.2) | 1520 (4.4) |  | 260 (3.2) | 231 (2.9) |  | 260 (3.2) | 1053 (2.6) |  |
| **IMD decile** |  |  |  |  |  | **0.053** |  |  | **0.042** |
| 1 | 1691 (21.1) | 6969 (20.1) | 0.102 | 1691 (21.1) | 1570 (19.6) |  | 1691 (21.1) | 8062 (20.2) |  |
| 2 | 973 (12.2) | 4912 (14.2) |  | 973 (12.2) | 1010 (12.6) |  | 973 (12.2) | 5084 (12.7) |  |
| 3 | 899 (11.2) | 4001 (11.6) |  | 899 (11.2) | 852 (10.6) |  | 899 (11.2) | 4362 (10.9) |  |
| 4 | 621 (7.8) | 2817 (8.1) |  | 621 (7.8) | 640 (8.0) |  | 621 (7.8) | 3170 (7.9) |  |
| 5 | 591 (7.4) | 2863 (8.3) |  | 591 (7.4) | 605 (7.6) |  | 591 (7.4) | 3074 (7.7) |  |
| 6 | 456 (5.7) | 2180 (6.3) |  | 456 (5.7) | 473 (5.9) |  | 456 (5.7) | 2472 (6.2) |  |
| 7 | 645 (8.1) | 2832 (8.2) |  | 645 (8.1) | 709 (8.9) |  | 645 (8.1) | 3403 (8.5) |  |
| 8 | 826 (10.3) | 3300 (9.5) |  | 826 (10.3) | 836 (10.4) |  | 826 (10.3) | 4015 (10.0) |  |
| 9 | 753 (9.4) | 2674 (7.7) |  | 753 (9.4) | 732 (9.1) |  | 753 (9.4) | 3644 (9.1) |  |
| 10 | 546 (6.8) | 2088 (6.0) |  | 546 (6.8) | 574 (7.2) |  | 546 (6.8) | 2719 (6.8) |  |
| **Ethnicity** |  |  | 0.11 |  |  | **0.032** |  |  | **0.064** |
| White | 6079 (76.0) | 27558 (79.6) |  | 6079 (76.0) | 6166 (77.1) |  | 6079 (76.0) | 31275 (78.2) |  |
| Asian or Asian British | 517 (6.5) | 1971 (5.7) |  | 517 (6.5) | 503 (6.3) |  | 517 (6.5) | 2513 (6.3) |  |
| Black or Black British | 317 (4.0) | 832 (2.4) |  | 317 (4.0) | 297 (3.7) |  | 317 (4.0) | 1592 (4.0) |  |
| Mixed | 68 (0.8) | 201 (0.6) |  | 68 (0.8) | 54 (0.7) |  | 68 (0.8) | 260 (0.6) |  |
| Other Ethnic Groups | 872 (10.9) | 3456 (10.0) |  | 872 (10.9) | 831 (10.4) |  | 872 (10.9) | 3743 (9.4) |  |
| Missing | 148 (1.8) | 618 (1.8) |  | 148 (1.8) | 150 (1.9) |  | 148 (1.8) | 622 (1.6) |  |
| **Diagnoses** |  |  |  |  |  |  |  |  |  |
| Diabetes | 2278 (28.5) | 10793 (31.2) | **0.059** | 2278 (28.5) | 2178 (27.2) | **0.028** | 2278 (28.5) | 10835 (27.1) | **0.031** |
| Hypertension | 4803 (60.0) | 25019 (72.2) | 0.26 | 4803 (60.0) | 4846 (60.6) | **0.011** | 4803 (60.0) | 24236 (60.6) | **0.011** |
| SLE | 13 (0.2) | 116 (0.3) | **0.035** | 13 (0.2) | 18 (0.2) | **0.014** | 13 (0.2) | 52 (0.1) | **0.021** |
| Gout | 946 (11.8) | 4837 (14.0) | **0.064** | 946 (11.8) | 927 (11.6) | **0.007** | 946 (11.8) | 4462 (11.2) | **0.016** |
| NAFLD | 126 (1.6) | 535 (1.5) | **0.002** | 126 (1.6) | 106 (1.3) | **0.021** | 126 (1.6) | 554 (1.4) | **0.014** |
| Myeloma | 15 (0.2) | 80 (0.2) | **0.01** | 15 (0.2) | 8 (0.1) | **0.023** | 15 (0.2) | 53 (0.1) | **0.013** |
| Osteoporosis | 341 (4.3) | 3157 (9.1) | 0.195 | 341 (4.3) | 348 (4.3) | **0.004** | 341 (4.3) | 1602 (4.0) | **0.039** |
| CAD | 1719 (21.5) | 7769 (22.4) | **0.023** | 1719 (21.5) | 1676 (20.9) | **0.013** | 1719 (21.5) | 7956 (19.9) | **0.046** |
| Heart failure | 605 (7.6) | 2986 (8.6) | **0.039** | 605 (7.6) | 548 (6.8) | **0.028** | 605 (7.6) | 2553 (6.4) | **0.009** |
| PAD | 313 (3.9) | 1703 (4.9) | **0.045** | 313 (3.9) | 266 (3.3) | **0.031** | 313 (3.9) | 1220 (3.0) | **0.047** |
| Stroke | 510 (6.4) | 2689 (7.8) | **0.054** | 510 (6.4) | 491 (6.1) | **0.01** | 510 (6.4) | 2175 (5.4) | **0.04** |
| TIA | 396 (4.9) | 2014 (5.8) | **0.038** | 396 (4.9) | 346 (4.3) | **0.03** | 396 (4.9) | 1649 (4.1) | **0.04** |
| ADPKD | 19 (0.2) | 566 (1.5) | 0.154 | 19 (0.2) | 20 (0.2) | **0.003** | 19 (0.2) | 87 (0.2) | **0.004** |
| Glomerulonephritis | 14 (0.2) | 159 (0.5) | **0.051** | 14 (0.2) | 12 (0.1) | **0.006** | 14 (0.2) | 66 (0.2) | **0.002** |
| Kidney stones | 187 (2.3) | 831 (2.4) | **0.004** | 187 (2.3) | 180 (2.2) | **0.006** | 187 (2.3) | 835 (2.1) | **0.017** |
| Vasculitis | 42 (0.5) | 250 (0.7) | **0.025** | 42 (0.5) | 32 (0.4) | **0.018** | 42 (0.5) | 191 (0.5) | **0.007** |
| Acute kidney injury | 821 (10.3) | 6290 (18.2) | 0.228 | 821 (10.3) | 738 (9.2) | **0.035** | 821 (10.3) | 3656 (9.1) | **0.038** |
| Depression | 2052 (25.6) | 10926 (31.5) | 0.131 | 2052 (25.6) | 1998 (25.0) | **0.016** | 2052 (25.6) | 9762 (24.4) | **0.029** |
| Schizophrenia | 179 (2.2) | 654 (1.9) | **0.052** | 179 (2.2) | 157 (2.0) | **0.019** | 179 (2.2) | 649 (1.6) | **0.045** |
| Bipolar disorder | 66 (0.8) | 302 (0.9) | **0.055** | 66 (0.8) | 51 (0.6) | **0.022** | 66 (0.8) | 231 (0.6) | **0.03** |
| Eating disorder | 96 (1.2) | 569 (1.6) | **0.077** | 96 (1.2) | 75 (0.9) | **0.026** | 96 (1.2) | 368 (0.9) | **0.027** |
| Self-harm and suicidal ideation | 218 (2.7) | 1026 (3.0) | **0.014** | 218 (2.7) | 222 (2.8) | **0.003** | 218 (2.7) | 896 (2.2) | **0.031** |
| Mean absolute SMD |  |  | 0.110 |  |  | 0.019 |  |  | 0.028 |
| **Key:** SMD = standardized mean difference. Excellent balance of covariates indicated by SMD value in bold i.e. <0.10. Mean absolute SMD values colour coded according to thresholds: red = poor balance; orange = moderate balance; green = excellent balance. | | | | | | | | | |

***CKD Stage 3: Risk of COVID-19 death – competing risk models accounting for risk of death***

- Patients were excluded that died before the beginning of the pandemic (t0 – 1^st^ March 2020).
- Across all competing risk models (table 4) coding status was not a significant predictor of a COVID-19 death when accounting for the competing risk of all-cause mortality. However, across models, the hazard ratio moved towards statistical significance (No PSM HR: 0.79, p=0.11; PSM 1:5 HR 0.77, p=0.07) with both hazard ratios similar negative values. Across all models, age groups 40-59, 75-89 and 90+ are all significant predictors of hazard of a COVID-19 death compared to age group 60-74. Across all models a one decile increase in IMD is associated with a lower hazard of a COVID-19 death. Specifically, for PSM model 1:5 it is associated with an 8% lower hazard of a COVID-19 death (HR 0.92, CIs 0.88-0.95, p=<0.0001). In PSM model 1:5, diabetes (HR 1.92, CIs 1.49-2.47, p=<0.0001), stroke (HR 1.97, CIs 1.41-2.75, p=<0.0001), CHD (HR 1.55, CIs 1.21-1.99, p= 0.0005), PAD (HR 1.91, CIs 1.41-2.75, p=0.001), and bipolar disorder (HR 6.96, CIs 3.36-14.41, p=<0.0001), were all associated with increased hazard of a COVID-19 death.

*Table 4: Competing risk regression models assessing risk of death in patients with coded and uncoded CKD stage 3 using, no PSM, PSM 1:1 and PSM 1:5 with replacement*

|  | **CKD Stage 3 - Risk of COVID-19 death accounting for competing risk of death** | | | | | | | | |
| --- | --- | --- | --- | --- | --- | --- | --- | --- | --- |
|  | **No PSM** | | | **PSM 1:1** | | | **PSM 1:5 w/ replacement** | | |
| **Variable** | **HR** | **P-value** | **95% CIs** | **HR** | **P-value** | **95% CIs** | **HR** | **P-value** | **95% CIs** |
| **Coded CKD** | 0.79 | 0.11 | 0.59-1.05 | 0.86 | 0.42 | 0.59-1.23 | 0.77 | 0.07 | 0.58-1.02 |
| **Male Sex** | 1.08 | 0.55 | 0.84-1.37 | 1.02 | 0.93 | 0.65-1.59 | 0.88 | 0.38 | 0.66-1.17 |
| **Age group 18-39** | 0.96 | 0.97 | 0.13-6.89 | 3x10^-4^ | **<0.0001** | 2x10^-4^-6x10^-4^ | 3x10^-4^ | **<0.0001** | 2x10^-4^-4x10^-4^ |
| **Age group 40-59** | 0.23 | **0.004** | 0.08-0.63 | 0.22 | **0.03** | 0.05-0.92 | 0.19 | **0.001** | 0.06-0.53 |
| **Age group 75-89** | 2.48 | **<0.0001** | 1.85-3.32 | 3.07 | **<0.0001** | 1.93-4.88 | 2.86 | **<0.0001** | 2.11-3.86 |
| **Age group 90+** | 5.57 | **<0.0001** | 3.70-8.39 | 8.39 | **<0.0001** | 4.13-17.04 | 9.44 | **<0.0001** | 6.18-14.41 |
| **BMI <18.5** | 1.91 | 0.06 | 0.98-3.73 | 1.49 | 0.59 | 0.35-6.30 | 2.47 | **0.01** | 1.21-5.02 |
| **BMI 25-29.9** | 0.97 | 0.83 | 0.73-1.29 | 0.97 | 0.89 | 0.60-1.55 | 1.04 | 0.79 | 0.78-1.37 |
| **BMI 30-39.9** | 1.08 | 0.62 | 0.80-1.45 | 1.14 | 0.62 | 0.68-1.91 | 0.87 | 0.40 | 0.62-1.20 |
| **BMI >=40** | 1.26 | 0.45 | 0.67-2.29 | 2.91 | **0.02** | 1.23-6.85 | 1.73 | 0.09 | 0.91-3.29 |
| **IMD** | 0.93 | **0.0005** | 0.89-0.97 | 0.95 | 0.11 | 0.90-1.01 | 0.92 | **<0.0001** | 0.88-0.95 |
| **Diabetes** | 1.74 | **<0.0001** | 1.38-2.18 | 2.37 | **<0.0001** | 1.60-3.50 | 1.92 | **<0.0001** | 1.49-2.47 |
| **Stroke** | 1.74 | **0.0005** | 1.27-2.38 | 1.85 | **<0.0001** | 1.07-3.16 | 1.97 | **<0.0001** | 1.41-2.75 |
| **CHD** | - | - | - | - | - | - | 1.55 | **0.0005** | 1.21-1.99 |
| **PAD** | - | - | - | - | - | - | 1.91 | **0.001** | 1.41-2.75 |
| **Bipolar disorder** | - | - | - | - | - | - | 6.96 | **<0.0001** | 3.36-14.41 |
| **Reference categories** | Coding status: uncoded at study start; Sex: Female; Age group: 40-59; BMI: 18.5-24.9 (healthy weight); Comorbidities: absence of diagnosis at study starts | | | | | | | | |
| **Statistically significant** | P-values in bold | | | | | | | | |
| **Not statistically significant** | Cells including - | | | | | | | | |

***CKD Stage 3: Impact of coding and COVID-19 vaccination on risk of COVID-19 Death – Propensity Score Matching***

- There is excellent balance for many individual diagnoses with moderate balance overall before matching (table 5). PSM 1:1 and 1:5 with replacement both offer excellent balance of covariates between coded and uncoded CKD stage 3.

*Table 5: PSM for patients with CKD stage 3 including vaccination status using 1:1 matching, and 1:5 matching with replacement: CRR models*

|  | **Before Matching** | | | **After Matching 1:1** | | | **After Matching 1:5 w/ replacement** | | |
| --- | --- | --- | --- | --- | --- | --- | --- | --- | --- |
|  | **Uncoded** | **Coded** | **SMD** | **Uncoded** | **Coded** | **SMD** | **Uncoded** | **Coded** | **SMD** |
| n | 6533 | 27821 |  | 6533 | 6533 |  | 6533 | 32665 |  |
| **Sex = M (%)** | 5453 (83.5) | 13208 (47.5) | 0.818 | 5453 (83.5) | 5449 (83.4) | **0.002** | 5453 (83.5) | 27184 (83.2) | **0.007** |
| **Age group (%)** |  |  | 0.344 |  |  | **0.054** |  |  | **0.036** |
| 18-39 | 61 (0.9) | 209 (0.8) |  | 61 (0.9) | 41 (0.6) |  | 61 (0.9) | 213 (0.7) |  |
| 40-59 | 1115 (17.1) | 2729 (9.8) |  | 1115 (17.1) | 1022 (15.6) |  | 1115 (17.1) | 5394 (16.5) |  |
| 60-74 | 3111 (47.6) | 10971 (39.4) |  | 3111 (47.6) | 3207 (49.1) |  | 3111 (47.6) | 15701 (48.1) |  |
| 75-89 | 2157 (33.0) | 13139 (47.2) |  | 2157 (33.0) | 2178 (33.3) |  | 2157 (33.0) | 10913 (33.4) |  |
| 90+ | 89 (1.4) | 773 (2.8) |  | 89 (1.4) | 85 (1.3) |  | 89 (1.4) | 444 (1.4) |  |
| **BMI group** |  |  | 0.102 |  |  | **0.013** |  |  | **0.029** |
| <18.5 | 33 (0.5) | 248 (0.9) |  | 33 (0.50 | 30 (0.5) |  | 33 (0.5) | 152 (0.5) |  |
| 18.5-24.9 | 1283 (19.6) | 5784 (20.8) |  | 1283 (19.6) | 1263 (19.3) |  | 1283 (19.6) | 6058 (18.5) |  |
| 25-29.9 | 2811 (43.0) | 10940 (39.3) |  | 2811 (43.0) | 2837 (43.4) |  | 2811 (43.0) | 14243 (43.6) |  |
| 30-39.9 | 2195 (33.6) | 9608 (34.5) |  | 2195 (33.6) | 2185 (33.4) |  | 2195 (33.6) | 11129 (34.1) |  |
| >=40 | 211 (3.2) | 1241 (4.5) |  | 211 (3.2) | 218 (3.3) |  | 211 (3.2) | 1083 (3.3) |  |
| **IMD decile** |  |  | 0.089 |  |  | **0.034** |  |  | **0.038** |
| 1 | 1348 (20.6) | 5568 (20.0) |  | 1348 (20.6) | 1275 (19.5) |  | 1348 (20.6) | 6532 (20.0) |  |
| 2 | 778 (11.9) | 3755 (13.5) |  | 778 (11.9) | 797 (12.2) |  | 778 (11.9) | 3908 (12.0) |  |
| 3 | 729 (11.2) | 3120 (11.2) |  | 729 (11.2) | 711 (10.9) |  | 729 (11.2) | 3550 (10.9) |  |
| 4 | 503 (7.7) | 2256 (8.1) |  | 503 (7.7) | 520 (8.0) |  | 503 (7.7) | 2558 (7.8) |  |
| 5 | 488 (7.5) | 2295 (8.2) |  | 488 (7.5) | 484 (7.4) |  | 488 (7.5) | 2407 (7.4) |  |
| 6 | 385 (5.9) | 1803 (6.5) |  | 385 (5.9) | 401 (6.1) |  | 385 (5.9) | 2094 (6.4) |  |
| 7 | 538 (8.2) | 2320 (8.3) |  | 538 (8.2) | 563 (8.6) |  | 538 (8.2) | 2877 (8.8) |  |
| 8 | 672 (10.3) | 2725 (9.8) |  | 672 (10.3) | 579 (10.4) |  | 672 (10.3) | 3442 (10.5) |  |
| 9 | 631 (9.7) | 2246 (8.1) |  | 631 (9.7) | 636 (9.7) |  | 631 (9.7) | 3122 (9.6) |  |
| 10 | 461 (7.1) | 1733 (6.2) |  | 461 (7.1) | 467 (7.1) |  | 461 (7.1) | 2175 (6.7) |  |
| **Ethnicity** |  |  | 0.126 |  |  | **0.022** |  |  | **0.027** |
| White | 5113 (78.3) | 22935 (82.4) |  | 5113 (78.3) | 5128 (78.5) |  | 5113 (78.3) | 25861 (79.2) |  |
| Asian or Asian British | 446 (6.8) | 1700 (6.1) |  | 446 (6.8) | 453 (6.9) |  | 446 (6.8) | 2127 (6.5) |  |
| Black or Black British | 313 (4.8) | 790 (2.8) |  | 313 (4.8) | 288 (4.4) |  | 313 (4.8) | 1594 (4.9) |  |
| Mixed | 61 (0.9) | 180 (0.6) |  | 61 (0.9) | 56 (0.9) |  | 61 (0.9) | 285 (0.9) |  |
| Other Ethnic Groups | 477 (7.3) | 1732 (6.2) |  | 477 (7.3) | 489 (7.5) |  | 477 (7.3) | 2234 (6.4) |  |
| Missing | 123 (1.9) | 484 (1.7) |  | 123 (1.9) | 119 (1.8) |  | 123 (1.9) | 564 (1.7) |  |
| **Diagnoses** |  |  |  |  |  |  |  |  |  |
| Diabetes | 1750 (26.8) | 8304 (29.8) | **0.068** | 1750 (26.8) | 1684 (25.8) | **0.023** | 1750 (26.8) | 8381 (25.7) | **0.026** |
| Hypertension | 3804 (58.2) | 19700 (70.8) | 0.265 | 3804 (58.2) | 3854 (59.0) | **0.016** | 3804 (58.2) | 19195 (58.8) | **0.011** |
| SLE | 10 (0.2) | 92 (0.3) | **0.036** | 10 (0.2) | <5 (<1.0) | **0.034** | 10 (0.2) | 36 (0.1) | **0.012** |
| Gout | 782 (12.0) | 3791 (13.6) | **0.050** | 782 (12.0) | 792 (12.1) | **0.005** | 782 (12.0) | 3853 (11.8) | **0.005** |
| NAFLD | 106 (1.6) | 464 (1.7) | **0.004** | 106 (1.6) | 95 (1.5) | **0.014** | 106 (1.6) | 521 (1.6) | **0.002** |
| Myeloma | 10 (0.2) | 60 (0.2) | **0.015** | 10 (0.2) | 11 (0.2) | **0.004** | 10 (0.2) | 51 (0.2) | **0.001** |
| Osteoporosis | 220 (3.4) | 2227 (8.0) | 0.201 | 220 (3.4) | 212 (3.2) | **0.007** | 220 (3.4) | 1041 (3.2) | **0.010** |
| CHD | 1270 (19.4) | 5614 (20.2) | **0.019** | 1270 (19.4) | 1239 (19.0) | **0.012** | 1270 (19.4) | 6228 (19.1) | **0.009** |
| Heart failure | 396 (6.1) | 1906 (6.9) | **0.032** | 396 (6.1) | 356 (5.4) | **0.026** | 396 (6.1) | 1706 (5.2) | **0.036** |
| PAD | 212 (3.2) | 1086 (3.9) | **0.035** | 212 (3.2) | 204 (3.1) | **0.007** | 212 (3.2) | 892 (2.7) | **0.030** |
| Stroke | 354 (5.4) | 1828 (6.6) | **0.049** | 354 (5.4) | 327 (5.0) | **0.019** | 354 (5.4) | 1589 (4.9) | **0.025** |
| TIA | 274 (4.2) | 1383 (5.0) | **0.037** | 274 (4.2) | 241 (3.7) | **0.026** | 274 (4.2) | 1147 (3.5) | **0.035** |
| ADPKD | 18 (0.3) | 465 (1.7 | 0.143 | 18 (0.3) | 22 (0.3) | **0.011** | 18 (0.3) | 94 (0.3) | **0.002** |
| Glomerulonephritis | 14 (0.2) | 148 (0.5) | **0.052** | 14 (0.2) | 15 (0.2) | **0.003** | 14 (0.2) | 67 (0.2) | **0.002** |
| Kidney stones | 156 (2.4) | 680 (2.4) | **0.004** | 156 (2.4) | 120 (1.8) | **0.038** | 156 (2.4) | 649 (2.0) | **0.027** |
| Vasculitis | 34 (0.5) | 203 (0.7) | **0.027** | 34 (0.5) | 23 (0.4) | **0.026** | 34 (0.5) | 134 (0.4) | **0.016** |
| Acute kidney injury | 643 (9.8) | 4859 (17.5) | 0.223 | 643 (9.8) | 587 (9.0) | **0.029** | 643 (9.8) | 2974 (9.1) | **0.025** |
| Depression | 1659 (25.4) | 8735 (31.4) | 0.133 | 1659 (25.4) | 1618 (24.8) | **0.014** | 1659 (25.4) | 7876 (24.1) | **0.030** |
| Schizophrenia | 149 (2.3) | 501 (1.8) | **0.034** | 149 (2.3) | 121 (1.9) | **0.030** | 149 (2.3) | 602 (1.8) | **0.031** |
| Bipolar disorder | 56 (0.9) | 254 (0.9) | **0.006** | 56 (0.9) | 34 (0.5) | **0.041** | 56 (0.9) | 197 (0.6) | **0.030** |
| Eating disorder | 72 (1.1) | 394 (1.4) | **0.028** | 72 (1.1) | 57 (0.9) | **0.023** | 72 (1.1) | 306 (0.9) | **0.016** |
| Self-harm and SI | 183 (2.8) | 853 (3.1) | **0.080** | 183 (2.8) | 171 (2.6) | **0.011** | 183 (2.8) | 790 (2.4) | **0.024** |
| **COVID-19 Vaccinated** | 6263 (95.9) | 27074 (97.3) | **0.080** | 6263 (95.9) | 6291 (96.3) | **0.022** | 6263 (95.9) | 31475 (96.4) | **0.025** |
| Mean absolute SMD |  |  | 0.110 |  |  | **0.020** |  |  | **0.020** |
| **Key:** SMD = standardized mean difference. Excellent balance of covariates indicated by SMD value in bold i.e. <0.10. Mean absolute SMD values colour coded according to thresholds: red = poor balance; orange = moderate balance; green = excellent balance. | | | | | | | | | |

***CKD Stage 3: Risk of COVID-19 death – accounting for COVID-19 vaccination – using PSM***

- Patients were excluded that died before the first available COVID-19 vaccination date (t0 – 8^th^ December 2020) (table 6).
- In PSM 1:1 model (table 6), coded CKD was not a significant predictor of a COVID-19 death when controlling for COVID-19 vaccination status. However, in PSM 1:5 model, coded CKD was a significant predictor (HR 0.63, CIs 0.43-0.90, p=0.01). In both PSM models, COVID-19 vaccination was associated with ~85% significantly lower risk of a COVID-19 death (without accounting for the competing risk of death). In PSM 1:5 model, regarding COVID-19 death, low BMI was associated with an increased hazard (HR 1.34, CIs 1.45-12.99, p=0.008); a one decile increase in IMD was associated with 9% lower hazard (HR 0.91, CIs 0.85-0.97, p=0.006). In PSM model 1:5, diabetes (HR 1.91, CIs 1.26-2.90, p=<0.002), PAD (HR 2.50, CIs 1.20-5.21, p=0.01) and stroke (HR 2.82 CIs 1.63-4.87, p=<0.0002) were all associated with increased hazard of a COVID-19 death.

*Table 6: Cox PH models assessing risk of COVID-19 death in patients with coded and uncoded CKD stage 3 with balancing of COVID-19 vaccination using PSM 1:1 and PSM 1:5 with replacement*

|  | **CKD Stage 3 – Impact of coding and COVID-19 vaccination on risk of COVID-19 death** | | | | | |
| --- | --- | --- | --- | --- | --- | --- |
|  | **PSM 1:1** | | | **PSM 1:5 w/ replacement** | | |
| **Variable** | **HR** | **P-value** | **95% CIs** | **HR** | **P-value** | **95% CIs** |
| **Coded CKD** | 0.72 | 0.13 | 0.49-1.10 | 0.63 | **0.01** | 0.43-0.90 |
| **COVID-19 vaccinated** | 0.18 | **<0.0001** | 0.10-0.32 | 0.14 | **<0.0001** | 0.08-0.26 |
| **Male Sex** | 1.00 | 0.99 | 0.61-1.65 | 0.89 | 0.60 | 0.57-1.39 |
| **BMI <18.5** | - | - | - | 4.34 | **0.008** | 1.45-12.99 |
| **BMI 25-29.9** | - | - | - | 1.14 | 0.60 | 0.69-1.88 |
| **BMI 30-39.9** | - | - | - | 1.38 | 0.21 | 0.83-2.27 |
| **BMI >= 40** | - | - | - | 1.27 | 0.61 | 0.50-3.23 |
| **IMD** | 0.93 | **0.04** | 0.87-0.99 | 0.91 | **0.006** | 0.85-0.97 |
| **Diabetes** | 2.07 | **0.0006** | 1.36-3.12 | 1.91 | **0.002** | 1.26-2.90 |
| **PAD** | 2.66 | **0.004** | 1.37-5.18 | 2.50 | **0.01** | 1.20-5.21 |
| **Stroke** | 2.86 | **<0.0001** | 1.69-4.84 | 2.82 | **0.0002** | 1.63-4.87 |
| **Reference categories** | Coding status: uncoded at study start; Sex: Female; Age group: 40-59; BMI: 18.5-24.9 (healthy weight); Comorbidities: absence of diagnosis at study starts | | | | | |
| **Statistically significant** | P-values in bold | | | | | |
| **Not statistically significant** | Cells including - | | | | | |
| **Stratified variables** | Ethnicity and age group violated the PH assumption. | | | | | |

***CKD Stage 4 Risk of Death: Propensity Score Matching***

There is significant imbalance before matching (table 7). After PSM 1:1, there is a slight improvement in balance additionally in variables diabetes and depression. After PSM 1:5 there is further slight improvement in balance additionally in hypertension, however there is residual imbalance in variables sex, age groups, BMI group, IMD deciles, and ethnicities. Overall, both PSM offer moderate overall balance.

*Table 7: PSM for patients with CKD stage 4 using 1:1 matching, and 1:5 matching with replacement.*

|  | **Before Matching** | | | **After Matching 1:1** | | | **After Matching 1:5 w/ replacement** | | |
| --- | --- | --- | --- | --- | --- | --- | --- | --- | --- |
|  | **Uncoded** | **Coded** | **SMD** | **Uncoded** | **Coded** | **SMD** | **Uncoded** | **Coded** | **SMD** |
| n | 75 | 3451 |  | 69 | 69 |  | 69 | 340 |  |
| **Sex = M (%)** | 60 (80.0) | 2347 (68.0) | 0.28 | 54 (78.3) | 51 (73.9) | 0.102 | 54 (78.3) | 274 (80.6) | 0.06 |
| **Age group (%)** |  |  | 0.37 |  |  | 0.209 |  |  | 0.20 |
| 18-39 | <5 (<3.0) | 48 (1.4) |  | <5 (<3.0) | <5 (<3.0) |  | <5 (<3.0) | 6 (1.8) |  |
| 40-59 | 6 (8.0) | 309 (9.0) |  | 6 (8.7) | 5 (7.2) |  | 6 (8.7) | 33 (9.7) |  |
| 60-74 | 16 (21.3) | 919 (26.6) |  | 16 (23.2) | 22 (31.9) |  | 16 (23.2) | 82 (24.1) |  |
| 75-89 | 35 (46.7) | 1868 (54.1) |  | 34 (49.3) | 32 (46.4) |  | 34 (49.3) | 159 (46.8) |  |
| 90+ | 16 (21.3) | 307 (8.9) |  | 13 (18.8) | 10 (14.5) |  | 13 (18.8) | 60 (17.6) |  |
| **BMI group** |  |  | 0.41 |  |  | 0.13 |  |  | 0.18 |
| <18.5 | <5 (<7.0) | 53 (1.5) |  | 5 (7.2) | <5 (<7.0) |  | 5 (7.2) | 12 (3.5) |  |
| 18.5-24.9 | 27 (36.0) | 841 (24.4) |  | 21 (30.4) | 21 (30.4) |  | 21 (30.4) | 117 (34.4) |  |
| 25-29.9 | 23 (30.7) | 1304 (37.8) |  | 23 (33.3) | 25 (36.2) |  | 23 (33.3) | 109 (32.1) |  |
| 30-39.9 | 16 (21.3) | 1074 (31.1) |  | 16 (23.2) | 16 (23.2) |  | 16 (23.2) | 83 (24.4) |  |
| >=40 | <5 (<7.0) | 179 (5.2) |  | <5 (<6.0) | <5 (<7.0) |  | <5 (<7.0) | 19 (5.6) |  |
| **IMD decile** |  |  | 0.28 |  |  | 0.31 |  |  | 0.39 |
| 1 | 12 (16.0) | 797 (23.1) |  | 11 (15.9) | 17 (24.6) |  | 11 (15.9) | 85 (25.0) |  |
| 2 | 14 (18.7) | 496 (14.4) |  | 11 (15.9) | 10 (14.5) |  | 11 (15.9) | 60 (17.6) |  |
| 3 | 9 (12.0) | 354 (10.3) |  | 9 (13.0) | 10 (14.5) |  | 9 (13.0) | 30 (8.8) |  |
| 4 | 9 (12.0) | 298 (8.6) |  | 9 (13.0) | 5 (7.2) |  | 9 (13.0) | 23 (6.8) |  |
| 5 | <5 (<6.0) | 254 (7.4) |  | <5 (<6.0) | 5 (7.2) |  | <5 (<6.0) | 20 (5.9) |  |
| 6 | 5 (6.7) | 187 (5.4) |  | <5 (<6.0) | 5 (7.2) |  | <5 (<6.0) | 15 (4.4) |  |
| 7 | 6 (8.0) | 299 (8.7) |  | 5 (7.2) | <5 (<6.0) |  | 5 (7.2) | 43 (12.6) |  |
| 8 | 9 (12.0) | 331 (9.6) |  | 9 (13.0) | 7 (10.1) |  | 9 (13.0) | 30 (8.8) |  |
| 9 | <5 (<6.0) | 267 (7.7) |  | <5 (<6.0) | <5 (<6.0) |  | <5 (<6.0) | 19 (5.6) |  |
| 10 | <5 (<6.0) | 168 (4.9) |  | <5 (<6.0) | <5 (<6.0) |  | <5 (<6.0) | 15 (4.4) |  |
| **Ethnicity** |  |  | 0.62 |  |  | 0.25 |  |  | 0.18 |
| White | 48 (64.0) | 2537 (73.5) |  | 48 (69.6) | 50 (72.5) |  | 48 (69.6) | 224 (65.9) |  |
| Asian or Asian British | <5 (<2.0) | 307 (8.9) |  | <5 (<2.0) | <5 (<2.0) |  | <5 (<2.0) | <5 (<2.0) |  |
| Black or Black British | <5 (<2.0) | 93 (2.7) |  | <5 (<2.0) | <5 (<2.0) |  | <5 (<2.0) | <5 (<2.0) |  |
| Mixed | <5 (<2.0) | 17 (0.5) |  | <5 (<2.0) | <5 (<2.0) |  | <5 (<2.0) | <5 (<2.0) |  |
| Other Ethnic Groups | 14 (18.7) | 417 (12.1) |  | 14 (20.3) | 13 (18.8) |  | 13 (18.8) | 85 (25.0) |  |
| Missing | 11 (14.7) | 80 (2.3) |  | 5 (7.2) | 6 (8.7) |  | 6 (8.7) | 26 (7.6) |  |
| **Diagnoses** |  |  |  |  |  |  |  |  |  |
| Diabetes | 20 (26.7) | 1654 (47.9) | 0.45 | 18 (26.1) | 20 (29.0) | **0.06** | 20 (29.0) | 104 (30.6) | **0.04** |
| Hypertension | 43 (57.3) | 2816 (81.6) | 0.55 | 38 (55.1) | 42 (60.9) | 0.12 | 42 (60.9) | 213 (62.6) | **0.04** |
| CHD | 20 (26.7) | 1038 (30.1) | **0.08** | 19 (27.5) | 19 (27.5) | **0.001** | 19 (27.5) | 89 (26.2) | **0.03** |
| Heart failure | 13 (17.3) | 561 (16.3) | **0.03** | 10 (14.5) | 12 (17.4) | **0.08** | 12 (17.4) | 46 (13.5) | **0.11** |
| Acute kidney injury | 16 (21.3) | 799 (23.2) | **0.04** | 19 (27.5) | 16 (23.2) | **0.10** | 16 (23.2) | 86 (25.3) | **0.05** |
| Depression | 16 (21.3) | 901 (26.1) | 0.11 | 17 (24.6) | 15 (21.7) | **0.07** | 15 (21.7) | 78 (22.9) | **0.03** |
| Mean absolute SMD |  |  | 0.290 |  |  | 0.132 |  |  | 0.120 |
| **Key:** SMD = standardized mean difference. Excellent balance of covariates indicated by SMD value in bold i.e. <0.10. Mean absolute SMD values colour coded according to thresholds: red = poor balance; orange = moderate balance; green = excellent balance. | | | | | | | | | |

***CKD Stage 4: Risk of Death – using PSM***

- In both models using PSM (table 8), coded CKD stage 4 (versus uncoded CKD stage 4) was associated with a consistently significantly lower hazard of death. Specifically, in PSM 1:1 model (table 8), coded CKD stage 4 was associated with 64% lower hazard of death (HR 0.34, CIs 0.20-0.60, p=0.0002); overweight BMI was associated with 61% lower hazard of death (HR 0.39, CIs 0.19-0.77); a one decile increase in IMD was associated with a 16% lower hazard of death (HR 0.84, CIs 0.76-0.94, p=0.003); and heart failure was associated with 83% increased hazard of death (HR 3.36, CIs 1.76-6.31, p=0.0002). Specifically, in PSM 1:5 model (table 8), coded CKD stage 4 was associated with 54% lower hazard of death (HR 0.46, CIs 0.32-0.66, p=<0.0001); a one decile increase in IMD was associated with a 8% lower hazard of death (HR 0.92, CIs 0.86-0.97, p=0.002); and heart failure was associated with 54% increased hazard of death (HR 1.54, CIs 1.08-2.20, p=0.02).

*Table 8: Cox PH models assessing risk of death in patients with coded and uncoded CKD stage 4 using PSM 1:1 and PSM 1:5 with replacement*

|  | **CKD Stage 4 – Risk of Death** | | | | | |
| --- | --- | --- | --- | --- | --- | --- |
|  | **PSM 1:1** | | | **PSM 1:5 w/ replacement** | | |
| **Variable** | **HR** | **P-value** | **95% CIs** | **HR** | **P-value** | **95% CIs** |
| **Coded CKD** | 0.34 | **0.0002** | 0.20-0.60 | 0.46 | **<0.0001** | 0.32-0.66 |
| **Male Sex** | 1.18 | 0.60 | 0.65-2.14 | 0.77 | 0.14 | 0.54-1.09 |
| **BMI <18.5** | 0.34 | 0.09 | 0.10-1.16 | 1.13 | 0.74 | 0.54-1.10 |
| **BMI 25-29.9** | 0.39 | **0.006** | 0.19-0.77 | 0.84 | 0.34 | 0.60-1.19 |
| **BMI 30-39.9** | 0.86 | 0.70 | 0.42-1.77 | 1.27 | 0.23 | 0.86-1.87 |
| **BMI >=40** | 0.72 | 0.63 | 0.19-2.72 | 1.78 | 0.12 | 0.86-3.69 |
| **IMD decile** | 0.84 | **0.003** | 0.76-0.94 | 0.92 | **0.002** | 0.86-0.97 |
| **Heart failure** | 3.36 | **0.0002** | 1.78-6.31 | 1.54 | **0.02** | 1.08-2.20 |
| **Reference categories** | Coding status: uncoded at study start; Sex: Female; BMI: 18.5-24.9 (healthy weight); IMD: a one decile increase in IMD; Comorbidities: absence of diagnosis at study start. | | | | | |
| **Statistically significant** | P-values in bold | | | | | |
| **Not statistically significant** | Cells including - | | | | | |
| **Stratified variables** | Ethnicity as it violated the PH assumption | | | | | |

***CKD Stage 4 Risk of Death: Propensity Score Matching (with modified variables)***

- To improve upon the last modelling for CKD stage 4 risk of death with PSM (tables 7 and 8), the below PSM (table 9) uses a simplified set of covariates (age group, IMD, and ethnicity) to balance.
- There is significant imbalance before matching (table 9). Both PSM models create excellent balance.
- Modifying variables (age group to fewer categories, IMD deciles to quintiles and ethnicity to White and Non-White) improved balance in PSM 1:5 for age group.

*Table 9: PSM for patients with CKD stage 4 using 1:1 matching, and 1:5 matching with replacement.*

|  | **Before Matching** | | | **After Matching 1:1** | | | **After Matching 1:5 w/ replacement** | | |
| --- | --- | --- | --- | --- | --- | --- | --- | --- | --- |
|  | **Uncoded** | **Coded** | **SMD** | **Uncoded** | **Coded** | **SMD** | **Uncoded** | **Coded** | **SMD** |
| n | 75 | 3451 |  | 71 | 71 |  | 71 | 339 |  |
| **Sex = M (%)** | 60 (80.0) | 2347 (68.0) | 0.276 | 57 (80.3) | 58 (81.7) | **0.036** | 57 (80.3) | 276 (81.4) | **0.029** |
| **Age group (%)** |  |  | 0.125 |  |  |  |  |  |  |
| 18-59 | 8 (10.7) | 357 (10.3) |  | 6 (8.5) | <5 (<6.0) | 0.121 | 6 (8.5) | 37 (10.9) | **0.084** |
| 60-74 | 16 (21.3) | 919 (26.6) |  | 16 (22.5) | 18 (25.4) |  | 16 (22.5) | 73 (21.5) |  |
| 75+ | 51 (68.0) | 2175 (63.0) |  | 49 (69.0) | 49 (69.0) |  | 49 (69.0) | 229 (67.6) |  |
| **BMI group** |  |  | 0.409 |  |  | 0.209 |  |  | 0.154 |
| <18.5 | 5 (6.7) | 53 (1.5) |  | <5 (<6.0) | 3 (4.2) |  | <5 (<6.0) | 11 (3.2) |  |
| 18.5-24.9 | 27 (36.0) | 841 (24.4) |  | 24 (33.8) | 21 (29.6) |  | 24 (33.8) | 115 (33.9) |  |
| 25-29.9 | 23 (30.7) | 841 (24.4) |  | 23 (32.4) | 28 (39.4) |  | 23 (32.4) | 127 (37.5) |  |
| 30-39.9 | 16 (21.3) | 1074 (31.1) |  | 16 (22.5) | 17 (23.9) |  | 16 (22.5) | 70 (20.6) |  |
| >=40 | <5 (<6.0) | 179 (5.2) |  | <5 (<6.0) | 2 (2.8) |  | <5 (<6.0) | 16 (4.7) |  |
| **IMD quintiles** |  |  | 0.162 |  |  | 0.320 |  |  | 0.350 |
| 1 | 26 (34.7) | 1293 (37.5) |  | 23 (32.4) | 30 (42.3) |  | 23 (32.4) | 157 (46.3) |  |
| 2 | 18 (24.0) | 652 (18.9) |  | 17 (23.9) | 10 (14.1) |  | 17 (23.9) | 49 (14.5) |  |
| 3 | 9 (12.0) | 441 (12.8) |  | 9 (12.7) | 12 (16.9) |  | 9 (12.7) | 43 (12.7) |  |
| 4 | 15 (20.0) | 630 (18.3) |  | 15 (21.1) | 14 (19.7) |  | 15 (21.1) | 51 (15.0) |  |
| 5 | 7 (9.3) | 435 (12.6) |  | 7 (9.9) | 5 (7.0) |  | 7 (9.9) | 39 (11.5) |  |
| **Ethnicity** |  |  | 0.454 |  |  | 0.124 |  |  | 0.148 |
| White | 48 (64.0) | 2537 (73.5) |  | 47 (66.2) | 47 (66.2) |  | 47 (66.2) | 246 (72.6) |  |
| Non-White | 16 (21.3) | 834 (24.2) |  | 16 (22.5) | 13 (18.3) |  | 16 (22.5) | 66 (19.5) |  |
| Missing | 11 (14.7) | 80 (2.3) |  | 8 (11.3) | 10 (14.3) |  | 8 (11.3) | 27 (8.0) |  |
| **Diagnoses** |  |  |  |  |  |  |  |  |  |
| Diabetes | 20 (26.7) | 1654 (47.9) | 0.451 | 20 (28.2) | 22 (31.0) | **0.062** | 20 (28.2) | 96 (28.3) | **0.003** |
| Hypertension | 43 (57.3) | 2816 (81.6) | 0.546 | 43 (60.6) | 43 (60.6) | **<0.001** | 43 (60.6) | 203 (59.9) | **0.014** |
| CHD | 20 (26.7) | 1038 (30.1) | **0.076** | 20 (28.2) | 19 (26.8) | **0.032** | 20 (28.2) | 106 (31.3) | **0.068** |
| Heart failure | 13 (17.3) | 561 (16.3) | **0.029** | 12 (16.9) | 11 (15.5) | **0.038** | 12 (16.9) | 55 (16.2) | **0.018** |
| Acute kidney injury | 16 (21.3) | 799 (23.2) | **0.044** | 16 (22.5) | 16 (22.5) | **<0.001** | 16 (22.5) | 75 (22.1) | **0.010** |
| Depression | 16 (21.3) | 901 (26.1) | 0.112 | 15 (21.1) | 14 (19.7) | **0.035** | 15 (21.1) | 71 (20.9) | **0.004** |
| Mean absolute SMD |  |  | 0.244 |  |  | **0.089** |  |  | **0.080** |
| **Key:** SMD = standardized mean difference. Excellent balance of covariates indicated by SMD value in bold i.e. <0.10. Mean absolute SMD values colour coded according to thresholds: red = poor balance; orange = moderate balance; green = excellent balance. | | | | | | | | | |

***CKD Stage 4: Risk of Death – using PSM and a modified set of covariates***

- The aim of these PSM models (table 10) are to improve covariate balance by simplifying 3 categorical variables (age group, IMD decile, and ethnicity) between coded and uncoded groups. These simplified models achieve better balance than similar models in table 7.
- In both of these simplified PSM models, coded CKD stage 4 (versus uncoded CKD stage 4) remained associated with a significantly lower hazard of death. Specifically, in PSM 1:1 model, coded CKD stage 4 was associated with 69% lower hazard of death (HR 0.31, CIs 0.27-0.53, p=<0.0001). Specifically, in PSM 1:5 model, coded CKD stage 4 was associated with 42% lower hazard of death (HR 0.38, CIs 0.27-0.53, p=<0.0001).

*Table 10: Cox PH models assessing risk of death in patients with coded and uncoded CKD stage 4 using PSM 1:1 and PSM 1:5 with replacement (modified variables age group, IMD, and ethnicity)*

|  | **CKD Stage 4 – Risk of Death (with modified variables)** | | | | | |
| --- | --- | --- | --- | --- | --- | --- |
|  | **PSM 1:1** | | | **PSM 1:5 w/ replacement** | | |
| **Variable** | **HR** | **P-value** | **95% CIs** | **HR** | **P-value** | **95% CIs** |
| **Coded CKD** | 0.31 | **<0.0001** | 0.19-0.53 | 0.38 | **<0.0001** | 0.27-0.53 |
| **Male Sex** | 1.46 | 0.25 | 0.77-2.75 | 1.15 | 0.55 | 0.73-1.80 |
| **Age group 18-59** | 0.19 | 0.11 | 0.02-1.48 | 0.26 | **0.01** | 0.09-0.76 |
| **Age group 75+** | 2.19 | **0.03** | 1.08-4.41 | 2.41 | **0.0003** | 1.50-3.87 |
| **BMI <18.5** | 0.62 | 0.40 | 0.21-1.87 | 1.13 | 0.79 | 0.47-2.70 |
| **BMI 25-29.9** | 0.73 | 0.29 | 0.41-1.31 | 0.60 | **0.004** | 0.42-0.85 |
| **BMI 30-39.9** | 0.72 | 0.36 | 0.35-1.45 | 0.71 | 0.18 | 0.43-1.17 |
| **BMI >=40** | 1.06 | 0.93 | 0.29-3.83 | 1.08 | 0.89 | 0.38-3.07 |
| **IMD quintiles** | 0.81 | **0.02** | 0.68-0.97 | 0.90 | **0.05** | 0.81-1.00 |
| **Heart failure** | 1.72 | 0.07 | 0.95-3.11 | 1.93 | **0.0005** | 1.33-2.80 |
| **CHD** | 1.83 | **0.03** | 1.05-3.18 | - | - | - |
| **Reference categories** | Coding status: uncoded at study start; Sex: Female; Age group: 40-59; BMI: 18.5-24.9 (healthy weight); Comorbidities: absence of diagnosis at study starts | | | | | |
| **Statistically significant** | P-values in bold | | | | | |
| **Not statistically significant** | Cells including - | | | | | |
| **Stratified variables** | Ethnicity as it violated the PH assumption | | | | | |

***CKD Stage 4 Risk of COVID-19 death competing risk models with risk of death: Propensity Score Matching***

- There is significant imbalance before matching (table 11). PSM 1:1 and 1:5 with replacement both offer overall excellent balance of covariates between coded and uncoded CKD stage 3.

*Table 11: PSM for patients with CKD stage 3 including vaccination status using 1:1 matching, and 1:5 matching with replacement: CRR models*

|  | **Before Matching** | | | **After Matching 1:1** | | | **After Matching 1:5 w/ replacement** | | |
| --- | --- | --- | --- | --- | --- | --- | --- | --- | --- |
|  | **Uncoded** | **Coded** | **SMD** | **Uncoded** | **Coded** | **SMD** | **Uncoded** | **Coded** | **SMD** |
| n | 528 | 2965 |  | 513 | 513 |  | 524 | 2603 |  |
| **Sex = M (%)** | 429 (81.2) | 2125 (71.7) | 0.227 | 415 (80.9) | 426 (83.0) | **0.056** | 425 (81.1) | 2114 (81.2) | **0.003** |
| **Age group (%)** |  |  | 0.436 |  |  | **0.065** |  |  | **0.037** |
| 18-39 | 20 (3.8) | 33 (1.1) |  | 15 (2.9) | 15 (2.9) |  | 19 (3.6) | 98 (3.6) |  |
| 40-59 | 78 (14.8) | 212 (7.2) |  | 72 (14.0) | 61 (11.9) |  | 75 (14.3) | 340 (13.1) |  |
| 60-74 | 175 (33.1) | 732 (24.7) |  | 171 (33.3) | 178 (34.7) |  | 175 (33.4) | 887 (34.1) |  |
| 75-89 | 234 (44.3) | 1727 (58.2) |  | 234 (45.6) | 238 (46.4) |  | 234 (44.7) | 1171 (45.0) |  |
| 90+ | 21 (4.0) | 261 (8.8) |  | 21 (4.1) | 21 (4.1) |  | 21 (4.0) | 107 (4.1) |  |
| **BMI group** |  |  | 0.183 |  |  | **0.053** |  |  | **0.042** |
| <18.5 | 9 (1.7) | 28 (0.9) |  | 7 (1.4) | 6 (1.2) |  | 7 (1.3) | 35 (1.3) |  |
| 18.5-24.9 | 162 (30.7) | 719 (24.2) |  | 154 (30.0) | 157 (30.6) |  | 162 (30.9) | 813 (31.2) |  |
| 25-29.9 | 173 (32.8) | 1161 (39.2) |  | 172 (33.5) | 179 (34.9) |  | 172 (32.8) | 894 (34.3) |  |
| 30-39.9 | 155 (29.4) | 920 (31.0) |  | 151 (29.4) | 140 (27.3) |  | 154 (29.4) | 721 (27.7) |  |
| >=40 |  |  |  | 29 (5.7) | 31 (6.0) |  |  |  |  |
| **IMD decile** |  |  | 0.165 |  |  | 0.140 |  |  | 0.140 |
| 1 | 116 (22.0) | 652 (22.0) |  | 112 (21.8) | 120 (23.4) |  | 114 (21.8) | 561 (21.6) |  |
| 2 | 91 (17.2) | 435 (14.7) |  | 88 (17.2) | 120 (23.4) |  | 90 (17.2) | 408 (15.7) |  |
| 3 | 55 (10.4) | 313 (10.6) |  | 53 (10.3) | 48 (9.4) |  | 55 (10.5) | 288 (11.1) |  |
| 4 | 39 (7.4) | 258 (8.7) |  | 38 (7.4) | 43 (8.4) |  | 39 (7.4) | 223 (8.6) |  |
| 5 | 26 (4.9) | 235 (7.9) |  | 26 (5.1) | 31 (6.0) |  | 26 (5.0) | 179 (6.9) |  |
| 6 | 36 (6.8) | 173 (5.8) |  | 35 (6.8) | 27 (5.3) |  | 36 (6.9) | 126 (4.8) |  |
| 7 | 48 (9.1) | 252 (8.5) |  | 47 (9.2) | 52 (10.1) |  | 47 (9.0) | 245 (9.4) |  |
| 8 | 57 (10.8) | 279 (9.4) |  | 54 (10.5) | 63 (12.3) |  | 57 (10.9) | 248 (9.5) |  |
| 9 | 33 (6.2) | 225 (7.6) |  | 33 (6.4) | 30 (5.8) |  | 33 (6.3) | 183 (7.0) |  |
| 10 | 27 (5.1) | 143 (4.8) |  | 27 (5.3) | 27 (5.3) |  | 27 (5.2) | 142 (5.5) |  |
| **Ethnicity** |  |  | 0.102 |  |  | **0.066** |  |  | **0.048** |
| White | 386 (73.1) | 2226 (75.1) |  | 375 (73.1) | 386 (75.2) |  | 385 (73.5) | 1937 (74.4) |  |
| Asian or Asian British | 47 (8.9) | 229 (7.7) |  | 45 (8.8) | 45 (8.8) |  | 46 (8.8) | 222 (8.5) |  |
| Black or Black British | 15 (2.8) | 69 (2.3) |  | 15 (2.9) | 14 (2.7) |  | 15 (2.9) | 60 (2.3) |  |
| Mixed | 6 (1.1) | 20 (0.7) |  | 6 (1.2) | 5 (1.0) |  | 6 (1.1) | 24 (0.9) |  |
| Other Ethnic Groups | 58 (11.0) | 360 (12.1) |  | 57 (11.1) | 52 (10.1) |  | 57 (10.9) | 277 (10.6) |  |
| Missing | 16 (3.0) | 61 (2.1) |  | 15 (2.9) | 11 (2.1) |  | 15 (2.9) | 83 (3.2) |  |
| **Diagnoses** |  |  |  |  |  |  |  |  |  |
| Diabetes | 206 (39.0) | 1439 (48.5) | 0.193 | 203 (39.6) | 204 (39.8) | **0.004** | 206 (39.3) | 1022 (39.3) | **0.001** |
| Hypertension | 299 (56.6) | 2406 (81.1) | 0.549 | 299 (58.3) | 302 (58.9) | **0.012** | 299 (57.1) | 1499 (57.6) | **0.011** |
| CHD | 111 (21.0) | 948 (32.0) | 0.250 | 111 (21.6) | 101 (19.7) | **0.048** | 11 (21.2) | 534 (20.5) | **0.016** |
| Heart failure | 70 (13.3) | 486 (16.4) | **0.088** | 70 (13.6) | 72 (14.0) | **0.011** | 70 (13.4) | 378 (14.5) | **0.034** |
| Stroke | 29 (5.5) | 283 (9.5) | 0.154 | 29 (5.7) | 33 (6.4) | **0.033** | 29 (5.5) | 167 (6.4) | **0.037** |
| Acute kidney injury | 90 (17.0) | 542 (18.3) | **0.032** | 90 (17.5) | 87 (17.0) | **0.015** | 90 (17.2) | 417 (16.0) | **0.031** |
| Depression | 123 (23.3) | 742 (25.0) | **0.040** | 120 (23.4) | 118 (23.0) | **0.009** | 123 (23.5) | 625 (24.0) | **0.013** |
| Mean absolute SMD |  |  | 0.201 |  |  | 0.048 |  |  | 0.034 |
| **Key:** SMD = standardized mean difference. Excellent balance of covariates indicated by SMD value in bold i.e. <0.10. Mean absolute SMD values colour coded according to thresholds: red = poor balance; orange = moderate balance; green = excellent balance. | | | | | | | | | |

***CKD Stage 4: Risk of COVID-19 death – competing risk models with risk of death - using PSM***

- In the ‘No PSM’ model, coded CKD stage was not associated with a significantly lower hazard of COVID-19 death accounting for the competing risk of death. However, both PSM models were associated with a significantly lower hazard of a COVID-19 death (PSM 1:1 HR 0.30, CIs 0.10-0.89, p=0.03; PSM 1:5 HR 0.50, CIs 0.27-0.93, p=0.03) after accounting for the competing risk of death. In PSM 1:5 model, additional predictors include overweight BMI (HR 3.42, CIs 1.49-7.84, p=0.004), depression (HR 1.99, CIs 1.03-3.80, p=0.04), and AKI (HR 1.96, CIs 1.02-3.77, p=0.04).

*Table 12: Competing risk regression models assessing risk of death in patients with coded and uncoded CKD stage 3 using, no PSM, PSM 1:1 and PSM 1:5 with replacement.*

|  | **CKD Stage 4 - Risk of COVID-19 death accounting for competing risk of death** | | | | | | | | |
| --- | --- | --- | --- | --- | --- | --- | --- | --- | --- |
|  | **No PSM** | | | **PSM 1:1** | | | **PSM 1:5 w/ replacement** | | |
| **Variable** | **HR** | **P-value** | **95% CIs** | **HR** | **P-value** | **95% CIs** | **HR** | **P-value** | **95% CIs** |
| **Coded CKD** | 0.57 | 0.08 | 0.30-1.07 | 0.30 | **0.03** | 0.10-0.89 | 0.50 | **0.03** | 0.27-0.93 |
| **Male Sex** | 0.90 | 0.73 | 0.50-1.62 | 0.27 | **0.004** | 0.11-0.65 | 0.85 | 0.66 | 0.42-1.75 |
| **Age group 18-39** | 1.31 | 0.79 | 0.17-9.90 | - | - | - | 0.47 | 0.49 | 0.05-3.40 |
| **Age group 40-59** | 0.66 | 0.51 | 0.67-0.51 | - | - | - | 0.56 | 0.32 | 0.20-1.70 |
| **Age group 75-89** | 1.51 | 0.20 | 0.81-2.81 | - | - | - | 1.13 | 0.71 | 0.60-2.14 |
| **Age group 90+** | 1.93 | 0.18 | 0.74-5.05 | - | - | - | 1.47 | 0.57 | 0.39-5.57 |
| **BMI <18.5** | 0.98 | 0.35 | 0.34-20.80 | - | - | - | 3.00 | 0.33 | 0.33-27.75 |
| **BMI 25-29.9** | 2.44 | **0.02** | 1.15-5.17 | - | - | - | 3.42 | **0.004** | 1.49-7.84 |
| **BMI 30-39.9** | 2.45 | **0.03** | 1.12-5.36 | - | - | - | 2.03 | 0.14 | 0.80-5.16 |
| **BMI >=40** | 1.43 | 0.65 | 0.30-6.71 | - | - | - | 1.49 | 0.62 | 0.30-7.33 |
| **Depression** | - | - | - | - | - | - | 1.99 | **0.04** | 1.03-3.80 |
| **AKI** | - | - | - | - | - | - | 1.96 | **0.04** | 1.02-3.77 |
| **Reference categories** | Coding status: uncoded at study start; Sex: Female; Age group: 40-59; BMI: 18.5-24.9 (healthy weight); Comorbidities: absence of diagnosis at study starts | | | | | | | | |
| **Statistically significant** | P-values in bold | | | | | | | | |
| **Statistically non-significant** | Cells with - | | | | | | | | |

***CKD Stage 4: Impact of coding and COVID-19 vaccination on risk of COVID-19 death with Propensity Score Matching***

- These PSM models seek to balance COVID-19 vaccination status across coded and uncoded CKD stage 4 – an important modifier of risk of COVID-19 death.
- Significant imbalances were present before matching (table 13). Both PSM models offer excellent overall balance, with PSM 1:5 with replacement offering the best overall balance.

*Table 13: PSM for patients with CKD stage 4 using 1:1 matching, and 1:5 matching with replacement.*

|  | **Before Matching** | | | **After Matching 1:1** | | | **After Matching 1:5 w/ replacement** | | |
| --- | --- | --- | --- | --- | --- | --- | --- | --- | --- |
|  | **Uncoded** | **Coded** | **SMD** | **Uncoded** | **Coded** | **SMD** | **Uncoded** | **Coded** | **SMD** |
| n | 353 | 1874 |  | 338 | 338 |  | 349 | 1707 |  |
| **Sex = M (%)** | 285 (80.7) | 1386 (74.0) | 0.162 | 270 (79.9) | 278 (82.2) | **0.060** | 281 (80.5) | 1398 (81.9) | **0.035** |
| **Age group (%)** |  |  | 0.429 |  |  | **0.126** |  |  | **0.094** |
| 18-39 | 19 (5.4) | 30 (1.6) |  | 16 (4.7) | 17 (15.0) |  | 18 (5.2) | 61 (3.6) |  |
| 40-59 | 68 (19.3) | 197 (10.5) |  | 57 (16.9 | 63 (18.6) |  | 66 (18.9) | 306 (17.9) |  |
| 60-74 | 129 (36.5) | 572 (30.5) |  | 118 (34.9) | 121 (35.8) |  | 128 (36.7) | 639 (37.4) |  |
| 75-89 | 126 (35.7) | 1001 (53.4) |  | 141 (41.7) | 126 (37.3) |  | 126 (36.1) | 656 (38.4) |  |
| 90+ | 11 (3.1) | 74 (3.9) |  | 6 (1.8) | 11 (3.3) |  | 11 (3.2) | 45 (2.6) |  |
| **BMI group** |  |  | 0.211 |  |  | 0.106 |  |  | **0.068** |
| <18.5 | 6 (1.7) | 11 (0.6) |  | 5 (1.5) | <5 (<1.0) |  | 5 (1.4) | 17 (1.0) |  |
| 18.5-24.9 | 93 (26.3) | 366 (19.5 |  | 88 (26.0) | 102 (30.2) |  | 91 (26.1) | 441 (25.8) |  |
| 25-29.9 | 122 (34.6) | 767 (40.9) |  | 119 (35.2) | 115 (34.0) |  | 121 (34.7) | 633 (37.1) |  |
| 30-39.9 | 133 (32.0) | 640 (34.2) |  | 108 (32.0) | 102 (30.2) |  | 113 (32.4) | 536 (31.4) |  |
| >=40 | 19 (5.4) | 90 (4.8) |  | 18 (5.3) | 16 (4.7) |  | 19 (5.4) | 80 (4.7) |  |
| **IMD decile** |  |  | 0.167 |  |  | 0.146 |  |  | 0.233 |
| 1 | 84 (23.8) | 418 (22.3) |  | 80 (23.7) | 80 (23.7) |  | 82 (23.5) | 351 (20.6) |  |
| 2 | 52 (14.7) | 248 (13.2) |  | 49 (14.5) | 46 (13.6) |  | 52 (14.9 | 220 (12.9) |  |
| 3 | 34 (9.6) | 177 (9.4) |  | 33 (9.8) | 27 (8.0) |  | 34 (9.7) | 177 (10.4) |  |
| 4 | 29 (8.2) | 162 (8.6) |  | 26 (7.7) | 25 (7.4) |  | 28 (8.0) | 163 (9.5) |  |
| 5 | 15 (4.2) | 145 (7.7) |  | 15 (4.4) | 23 (6.8) |  | 15 (4.3) | 149 (8.7) |  |
| 6 | 26 (7.4) | 110 (5.9) |  | 25 (7.4) | 22 (6.5) |  | 26 (7.4) | 83 (4.9) |  |
| 7 | 31 (8.8) | 158 (8.4) |  | 30 (8.9) | 30 (8.9) |  | 31 (8.9) | 169 (9.9) |  |
| 8 | 39 (11.0) | 208 (11.1) |  | 37 (10.9) | 38 (11.2) |  | 38 (10.9) | 173 (10.1) |  |
| 9 | 26 (7.4) | 153 (8.2) |  | 26 (7.7) | 24 (7.1) |  | 26 (7.4) | 127 (7.4) |  |
| 10 | 17 (4.8) | 95 (5.1) |  | 17 (5.0) | 23 (6.8) |  | 17 (4.9) | 95 (5.6) |  |
| **Ethnicity** |  |  | **0.089** |  |  | 0.106 |  |  | **0.064** |
| White | 277 (78.5) | 1484 (79.2) |  | 266 (78.7) | 273 (80.8) |  | 274 (78.5) | 1382 (81.0) |  |
| Asian or Asian British | 33 (9.3) | 161 (8.6) |  | 29 (8.6) | 32 (9.5) |  | 32 (9.2) | 12 (8.3) |  |
| Black or Black British | 14 (4.0) | 60 (3.2) |  | 14 (4.1) | 12 (3.6) |  | 14 (4.0) | 61 (3.6) |  |
| Mixed | <5 (<2.0) | 16 (0.9) |  | <5 (<2.0) | <5 (<2.0) |  | <5 (<2.0) | 19 (1.1) |  |
| Other Ethnic Groups | 17 (4.8) | 118 (6.3) |  | 17 (5.0) | 13 (3.8) |  | 17 (4.9) | 69 (4.0) |  |
| Missing | 8 (2.3) | 35 (1.9) |  | 8 (2.4) | 6 (1.8) |  | 8 (2.3) | 34 (2.0) |  |
| **Diagnoses** |  |  |  |  |  |  |  |  |  |
| Diabetes | 124 (35.1) | 895 (47.8) | 0.259 | 122 (36.1) | 130 (38.5) | **0.049** | 123 (35.2) | 607 (35.6) | **0.007** |
| Hypertension | 188 (53.3) | 1500 (80.0) | 0.593 | 188 (55.6) | 185 (54.7) | **0.018** | 188 (53.9) | 927 (54.3) | **0.009** |
| CHD | 56 (15.9) | 500 (26.7) | 0.267 | 56 (16.6) | 51 (15.1) | **0.041** | 56 (16.6) | 262 (15.3) | **0.019** |
| Heart failure | 33 (9.3) | 228 (12.2) | **0.091** | 33 (9.8) | 32 (9.5) | **0.010** | 33 (9.5) | 169 (9.9) | **0.015** |
| Stroke | 11 (3.1) | 134 (7.2) | 0.184 | 11 (3.3) | 10 (3.0) | **0.017** | 63 (3.2) | 11 (3.2) | **0.030** |
| Acute kidney injury | 58 (16.4 | 322 (17.2) | **0.020** | 55 (16.3) | 52 (15.4) | **0.024** | 57 (16.3) | 269 (15.8) | **0.016** |
| Depression | 85 (24.1) | 444 (23.7 | **0.009** | 82 (24.3) | 73 (21.6) | **0.063** | 84 (24.1) | 411 (24.1) | **0.001** |
| **COVID-19 Vaccinated** | 337 (95.5) | 1841 (98.2) | 0.159 | 327 (96.7) | 328 (97.0) | **0.017** | 337 (96.6) | 1671 (97.9) | **0.081** |
| Mean absolute SMD |  |  | 0.203 |  |  | **0.060** |  |  | **0.051** |
| **Key:** SMD = standardized mean difference. Excellent balance of covariates indicated by SMD value in bold i.e. <0.10. Mean absolute SMD values colour coded according to thresholds: red = poor balance; orange = moderate balance; green = excellent balance. | | | | | | | | | |

***CKD Stage 4: Risk of COVID-19 death – accounting for COVID-19 vaccination – using PSM***

- Patients were excluded that died before the first available COVID-19 vaccination date (t0 – 8^th^ December 2020) (table 14).
- In PSM 1:1 model, coded CKD was associated with an 81% lower hazard of a COVID-19 death (HR 0.19, CIs 0.04-0.86, p=0.03) when controlling for COVID-19 vaccination status.
- A similar estimate was also seen in PSM 1:5 model, where coded CKD was associated with a 75% lower hazard of a COVID-19 death (HR 0.25, CIs 0.10-0.60, p=0.002). In PSM 1:5 model, heart failure was associated with ~4 times increased hazard of a COVID-19 death after controlling for COVID-19 vaccination status.
- In both PSM models, COVID-19 vaccination status was not a significant predictor of a COVID-19 death across patients with coded and uncoded CKD stage 4.

*Table 14: Cox PH models assessing risk of COVID-19 death in patients with coded and uncoded CKD stage 4 with balancing of COVID-19 vaccination using PSM 1:1 and PSM 1:5 with replacement*

|  | **CKD Stage 4 – Impact of coding and COVID-19 vaccination on risk of COVID-19 death** | | | | | |
| --- | --- | --- | --- | --- | --- | --- |
|  | **PSM 1:1** | | | **PSM 1:5 w/ replacement** | | |
| **Variable** | **HR** | **P-value** | **95% CIs** | **HR** | **P-value** | **95% CIs** |
| **Coded CKD** | 0.19 | **0.03** | 0.04-0.86 | 0.25 | **0.002** | 0.10-0.60 |
| **COVID-19 vaccinated** | 0.60 | 0.68 | 0.05-6.72 | 0.30 | 0.16 | 0.05-1.64 |
| **Male Sex** | 0.21 | **0.02** | 0.06-0.78 | 0.73 | 0.53 | 0.27-1.96 |
| **Heart failure** | 6.76 | **0.02** | 1.35-33.70 | 3.98 | **0.005** | 1.52-10.41 |
| **Reference categories** | Coding status: uncoded at study start; Sex: Female; Age group: 40-59; BMI: 18.5-24.9 (healthy weight); Comorbidities: absence of diagnosis at study starts | | | | | |
| **Statistically significant** | P-values in bold | | | | | |
| **Not statistically significant** | Cells including - | | | | | |
| **Stratified variables** | Ethnicity, age group and BMI group violated the PH assumption. | | | | | |
