# Supplementary material for: Quantifying the impact of clinical coding in chronic kidney disease on risk of death and COVID-19 death
Source: PLoS One. 2025 Oct 24;20(10):e0333881. doi: 10.1371/journal.pone.0333881 (PMC12551823; doi:10.1371/journal.pone.0333881)
Supplement: Attachment — Submitted filename: PONE-D-25-26228_review_20250729.docx [file pone.0333881.s004.docx]

Thank you for giving me the opportunity to review the manuscript. This is a retrospective cohort study of patients with stage 3–5 chronic kidney disease (CKD). The authors reported that uncoded CKD was associated with a higher risk of all-cause and coronavirus disease 2019 (COVID-19) mortality. Although this is a thought-provoking study, several concerns need to be addressed.

**Major comments**

1) My greatest concern is statistical analysis. The aim of this study was to investigate the association between coding of CKD and all-cause and COVID-19 mortality among patients with stage 3–5 CKD. Therefore, I would suggest that the authors include all patients with stage 3–5 CKD in the main analysis. After conducting this main analysis, the authors need to perform the same analysis by subgroups of CKD (stages 3 and 4 CKD).
2) Given that the aim of this study was to investigate the association between coding of CKD and mortality, I would suggest that the authors compare the two groups in Table 1: patients with coded CKD and those with uncoded CKD.
3) Considering the study design and limitations of this study, I would suggest that the authors town the Conclusion section.
4) I would suggest that the authors clearly describe the definition of confirmed SARS-CoV-2 infection. Were the patients diagnosed with COVID-19 based on positive PCR or antigen test results?
5) I would suggest adding discussions regarding the high proportion of coded CKD and the role of primary care physicians in England. Surprisingly, 81.1% of the patients were coded as having CKD in this study. A previous study using databases from five countries (France, Germany, Italy, Japan, and the United States) reported consistently high proportions of patients with undiagnosed stage 3 CKD [**Ref #1**]. This discrepancy can, at least in part, be attributed to the quality of primary care physicians in England. A previous study reported that preventing hospitalization is a key role for primary care physicians, even during the COVID-19 pandemic [**Ref #2**]. Primary care physicians play an important role in the field of nephrology. A cohort study in Canada reported that patients with stage 1–4 CKD treated by primary care physicians had a lower risk of hospitalization for heart failure and hyperkalemia than those who were not treated by primary care physicians [**Ref #3**]. Regarding infections, primary care physician-nephrologist collaboration was associated with a lower risk of infection-related hospitalization among patients with stage 5 CKD [**Ref #4**]. The above discussions on the role of primary care physicians will enhance the importance of this study.

**Ref #1** Tangri N, et al. Prevalence of undiagnosed stage 3 chronic kidney disease in France, Germany, Italy, Japan and the USA: results from the multinational observational REVEAL-CKD study. BMJ Open. 2023;13:e067386.

**Ref #2** Aoki T, et al. Impact of primary care attributes on hospitalization during the COVID-19 pandemic: a nationwide prospective cohort study in Japan. Ann Fam Med 2023;21:27–32.

**Ref #3** Wiebe N, et al. Potentially preventable hospitalization as a complication of CKD: a cohort study. Am J Kidney Dis 2014;64:230–238.

**Ref #4** Murakami M et al. Association between primary care physician–nephrologist collaboration and clinical outcomes in patients with stage 5 chronic kidney disease: a JOINT‑KD cohort study. J Nephrol 2025;38:1385–1394.

6) I would suggest adding a supplementary figure showing the flowchart of the study participants because only 47,628 patients with stage 3–5 CKD out of 2.45 million patients were included in the analysis.
7) I would suggest adding the causes of CKD to Table 1. It is unclear whether diseases, such as diabetes and hypertension, are the causes of CKD or comorbidities.

**Minor comments**

8) I would suggest that the authors clarify why patients who died due to non-COVID-19 were excluded from the analysis (page 6). In the Cox proportional hazards model, the patients who experienced such events are censored. However, in the Fine–Gray model, such events are treated as competing risk events.
9) I would suggest adding a supplementary Figure showing the flowchart of the study participants because only 47,628 patients with stage 3–5 CKD out of 2.45 million patients were included in the analysis.
10) I would suggest that the authors clearly describe whether the hazard ratio is unadjusted or adjusted throughout the manuscript. The term “hazard ratio” is ambiguous for readers to understand the results of this study.
11) In relation to the above comment, I would suggest that Tables 2 and 3 list the adjusted hazard ratios, 95% confidence intervals, and p-values in that order.
12) I would suggest that the authors revise the Descriptive analyses by CKD stage section. As mentioned above, descriptions according to the CKD stage are not necessary because this is a subgroup of study participants. Moreover, these sentences appear redundant.
13) I would suggest combining Figures 3 with 4, and 5 with 6. A Kaplan–Meier curve with an enlarged y-axis is commonly incorporated within the area of the original Kaplan–Meier curve.
14) I would suggest that the author suggests clearly stating the p-value (page 15). when the p-value is 0.05, please include three decimal places (e.g., 0.048).
15) I would suggest that the authors recheck the reference list. For example, #20 of Reference needs to be revised as follows:

BMC Nephrol 2025;26:1–10. → BMC Nephrol 2025;26:39.

16) I would suggest that the authors correct grammatical errors throughout the manuscript.
