# Supplementary material for: Quantifying the impact of clinical coding in chronic kidney disease on risk of death and COVID-19 death
Source: PLoS One. 2025 Oct 24;20(10):e0333881. doi: 10.1371/journal.pone.0333881 (PMC12551823; doi:10.1371/journal.pone.0333881)
Supplement: Attachment — Submitted filename: Response to reviewers.docx [file pone.0333881.s006.docx]

Dear Editorial Team and Peer-reviewers,

Thanks for your reviews of our paper. Your critical and thoughtful comments helped identify opportunities to strengthen our paper.

Please find our comments below and our revised manuscript attached.

Dr Stuart Stewart

On behalf of all authors

**Editorial comments**

When submitting your revision, we need you to address these additional requirements.

1. Please ensure that your manuscript meets PLOS ONE's style requirements, including those for file naming. The PLOS ONE style templates can be found at

<https://journals.plos.org/plosone/s/file?id=wjVg/PLOSOne_formatting_sample_main_body.pdf> and

[https://journals.plos.org/plosone/s/file?id=ba62/PLOSOne_formatting_sample_title_authors_affiliations.pdf](https://track.editorialmanager.com/CL0/https:%2F%2Fjournals.plos.org%2Fplosone%2Fs%2Ffile%3Fid=ba62%2FPLOSOne_formatting_sample_title_authors_affiliations.pdf/1/010f01985c760768-6f802238-46f3-4c23-81ce-fcc2a38f4845-000000/bB8WBHr3PxHXtSqVrCUBejdbYrfVmglJfYs_HJw1pbM=222).

**Our response:** Please see our updated manuscript.

2. We note that you have indicated that there are restrictions to data sharing for this study. For studies involving human research participant data or other sensitive data, we encourage authors to share de-identified or anonymized data. However, when data cannot be publicly shared for ethical reasons, we allow authors to make their data sets available upon request. For information on unacceptable data access restrictions, please see <http://journals.plos.org/plosone/s/data-availability#loc-unacceptable-data-access-restrictions>.

Before we proceed with your manuscript, please address the following prompts:

a) If there are ethical or legal restrictions on sharing a de-identified data set, please explain them in detail (e.g., data contain potentially identifying or sensitive patient information, data are owned by a third-party organization, etc.) and who has imposed them (e.g., a Research Ethics Committee or Institutional Review Board, etc.). Please also provide contact information for a data access committee, ethics committee, or other institutional body to which data requests may be sent.

**Our response:** The manuscript addresses this point however we have now explicitly highlighted that we do not have permission to permit access to use this data. See section “Availability of data and materials: We do not have permission to share or permit access to the data used in this study. Researchers can apply to use the same GMCR data through an application to the GMCR board at the following address: <https://healthinnovationmanchester.com/contact/> “

b) If there are no restrictions, please upload the minimal anonymized data set necessary to replicate your study findings to a stable, public repository and provide us with the relevant URLs, DOIs, or accession numbers. Please see <http://www.bmj.com/content/340/bmj.c181.long> for guidelines on how to de-identify and prepare clinical data for publication. For a list of recommended repositories, please see <https://journals.plos.org/plosone/s/recommended-repositories>. You also have the option of uploading the data as Supporting Information files, but we would recommend depositing data directly to a data repository if possible.

Please update your Data Availability statement in the submission form accordingly.

3. Thank you for stating the following in the Competing Interests section*:

“I have read the journal's policy and the authors of this manuscript have the following competing interests: SSt receives funding from Kidney Research UK; award number AHPF_001_20220705. PK received grant funding from CSL Vifor and Astellas, consulting fees from Astra Zeneca, Vifor, Unicyte and UCB, honoraria from Astra Zeneca, Pfizer, Pharmacosmos, Medice, GSK, Bayer and CSL Vifor. EK – None to declare. TB – No financial conflicts of interest; NHS England Think Kidneys Programme Board Member (2014–17); Royal College of General Practitioners’ AKI Clinical Champion (2017–20); NHSE Renal Services Transformation Programme Post‑AKI care Lead (2021–23); Specialist Committee Member for NICE AKI Quality Standard (QS76) (2022–23); Kidney Disease Improving Global Outcomes (KDIGO) AKI Guideline Work Group (2023‑To date). GT – none to declare. SSi received honoraria from AZ, Boehringer, Menarini, Novartis, GSK, CSL Vifor and Bayer.”

We note that one or more of the authors have an affiliation to the commercial funders of this research study

a. Please provide an amended Funding Statement declaring this commercial affiliation, as well as a statement regarding the Role of Funders in your study. If the funding organization did not play a role in the study design, data collection and analysis, decision to publish, or preparation of the manuscript and only provided financial support in the form of authors' salaries and/or research materials, please review your statements relating to the author contributions, and ensure you have specifically and accurately indicated the role(s) that these authors had in your study. You can update author roles in the Author Contributions section of the online submission form.

Please also include the following statement within your amended Funding Statement.

“The funder provided support in the form of salaries for authors [insert relevant initials], but did not have any additional role in the study design, data collection and analysis, decision to publish, or preparation of the manuscript. The specific roles of these authors are articulated in the ‘author contributions’ section.”

**Our response:**  **Please update our funding statement to:** “SSt receives doctoral research funding from Kidney Research UK (AHP_001_202207405). The funder provided support in the form of salaries for SSt, but did not have any additional role in the study design, data collection and analysis, decision to publish, or preparation of the manuscript. GT is funded by the National Institute for Health and Care Research (NIHR) Manchester Biomedical Research Centre (BRC) (NIHR203308). The funder provided support in the form of salaries for SSt, but did not have any additional role in the study design, data collection and analysis, decision to publish, or preparation of the manuscript. The specific roles of these authors are articulated in the ‘author contributions’ section.”

If your commercial affiliation did play a role in your study, please state and explain this role within your updated Funding Statement.

b. Please also provide an updated Competing Interests Statement declaring this commercial affiliation along with any other relevant declarations relating to employment, consultancy, patents, products in development, or marketed products, etc.

Within your Competing Interests Statement, please confirm that this commercial affiliation does not alter your adherence to all PLOS ONE policies on sharing data and materials by including the following statement: "This does not alter our adherence to  PLOS ONE policies on sharing data and materials.” (as detailed online in our guide for authors <http://journals.plos.org/plosone/s/competing-interests>). If this adherence statement is not accurate and  there are restrictions on sharing of data and/or materials, please state these. Please note that we cannot proceed with consideration of your article until this information has been declared.

**Our response: Please update our competing interests statement to: “**SSt receives funding for salary from Kidney Research UK; award number AHPF_001_20220705. PK received grant funding from CSL Vifor and Astellas, consulting fees from Astra Zeneca, Vifor, Unicyte and UCB, honoraria from Astra Zeneca, Pfizer, Pharmacosmos, Medice, GSK, Bayer and CSL Vifor. EK – None to declare. TB – No financial conflicts of interest; NHS England Think Kidneys Programme Board Member (2014–17); Royal College of General Practitioners’ AKI Clinical Champion (2017–20); NHSE Renal Services Transformation Programme Post‑AKI care Lead (2021–23); Specialist Committee Member for NICE AKI Quality Standard (QS76) (2022–23); Kidney Disease Improving Global Outcomes (KDIGO) AKI Guideline Work Group (2023‑To date). GT receives salary funding by the National Institute for Health and Care Research (NIHR) Manchester Biomedical Research Centre (BRC) (NIHR 203308). SSi has received speaker fees from AstraZeneca, Bayer, Chiesi, Sanofi-Genzyme, Novartis, CSL Vifor, GSK, Menarini, Medscape, and Boehringer-Ingelheim; and consulting fees from AstraZeneca, Novo Nordisk, Amicus, Stada, Santhera, Novartis, Bayer, Sanofi-Genzyme, Vifor Pharma, GSK, Inozyme Pharma, Sobi, Inozyme Pharma Inc., Pursepring and Boehringer-Ingelheim, and grant funding received from JnJ, AstraZeneca, CSL Vifor, Sanofi-Genzyme, and Novartis. This does not alter our adherence to  PLOS ONE policies on sharing data and materials.”

Please include both an updated Funding Statement and Competing Interests Statement in your cover letter. We will change the online submission form on your behalf.

4. Thank you for stating the following in the Acknowledgments Section of your manuscript:

“GT is funded by the National Institute for Health and Care Research (NIHR) Manchester Biomedical Research Centre (BRC) (NIHR203308).”

We note that you have provided additional information within the Acknowledgements Section that is not currently declared in your Funding Statement. Please note that funding information should not appear in the Acknowledgments section or other areas of your manuscript. We will only publish funding information present in the Funding Statement section of the online submission form.

Please remove any funding-related text from the manuscript and let us know how you would like to update your Funding Statement. Currently, your Funding Statement reads as follows:

“Dr Stuart Stewart receives doctoral research funding from Kidney Research UK (AHP_001_202207405). The funder of the study had no role in article design, analysis, or publication writing.”

Please include your amended statements within your cover letter; we will change the online submission form on your behalf.

**Our response:** Please see our update at the beginning of this document.

5. Your ethics statement should only appear in the Methods section of your manuscript. If your ethics statement is written in any section besides the Methods, please move it to the Methods section and delete it from any other section. Please ensure that your ethics statement is included in your manuscript, as the ethics statement entered into the online submission form will not be published alongside your manuscript.

**Our response:** Please see our updated manuscript.

6. If the reviewer comments include a recommendation to cite specific previously published works, please review and evaluate these publications to determine whether they are relevant and should be cited. There is no requirement to cite these works unless the editor has indicated otherwise.

**Our response:** Please see our updated manuscript.

Additional Editor Comments (if provided):

Dear Authors,
after careful evaluation and consideration of the reviewers' comments, we believe that your manuscript has potential but requires substantial revisions before it can be considered for publication. Therefore, we are inviting you to submit a major revision.
Please address all the reviewers' comments thoroughly in your revised manuscript and provide a detailed response to each point raised. This will help us and the reviewers assess how the concerns have been addressed.
We appreciate the value of your work and look forward to receiving your revised manuscript, along with your responses to the reviewers’ remarks.

**General Reviewers' comments:**

Reviewer's Responses to Questions

**Comments to the Author**

1. Is the manuscript technically sound, and do the data support the conclusions?

The manuscript must describe a technically sound piece of scientific research with data that supports the conclusions. Experiments must have been conducted rigorously, with appropriate controls, replication, and sample sizes. The conclusions must be drawn appropriately based on the data presented.

Reviewer #1: Yes

Reviewer #2: Yes

2. Has the statistical analysis been performed appropriately and rigorously?

Reviewer #1: Yes

Reviewer #2: No

3. Have the authors made all data underlying the findings in their manuscript fully available?

The [PLOS Data policy](http://track.editorialmanager.com/CL0/http:%2F%2Fwww.plosone.org%2Fstatic%2Fpolicies.action%23sharing/1/010f01985c760768-6f802238-46f3-4c23-81ce-fcc2a38f4845-000000/Cx-oGuuO_cs5zrJnr2TXoujkGQsa5Ezwb4GU7Qx-57Q=222) requires authors to make all data underlying the findings described in their manuscript fully available without restriction, with rare exception (please refer to the Data Availability Statement in the manuscript PDF file). The data should be provided as part of the manuscript or its supporting information, or deposited to a public repository. For example, in addition to summary statistics, the data points behind means, medians and variance measures should be available. If there are restrictions on publicly sharing data—e.g. participant privacy or use of data from a third party—those must be specified.

Reviewer #1: Yes

Reviewer #2: Yes

4. Is the manuscript presented in an intelligible fashion and written in standard English?

PLOS ONE does not copyedit accepted manuscripts, so the language in submitted articles must be clear, correct, and unambiguous. Any typographical or grammatical errors should be corrected at revision, so please note any specific errors here.

Reviewer #1: Yes

Reviewer #2: Yes

**Specific Reviewer Comments**

**Reviewer #1**

1. Originality
This is an original study as it highlights that CKD coding is associated with a downstream alert signal for patients, potentially regardless of the medical specialty they are subsequently seen by. It emphasizes associated comorbidities and the possible impact on the overall clinical evaluation of these patients.

2. Importance
In a well-structured and educated healthcare system, CKD coding is a key element for the systematic monitoring of these patients and enables proper, structured management. It also facilitates early detection and highlights potential complications in their disease progression.

4. Results
Regardless of the future healthcare setting or possible instability in patient care, CKD coding remains a crucial element that benefits both the medical system and the patients.

**Questions:
1. Has any analysis been conducted on the patient follow-up rate after CKD coding?**

**Our response:** Thank you for raising this point. In our study, patient follow-up was from 2018 to 2023, during which we examined outcomes of all-cause mortality and COVID-19 mortality among patients with coded and uncoded CKD. While our analyses incorporate longitudinal follow-up across this period, our primary focus was on mortality outcomes rather than follow-up after the uncoded cohort were coded.

**2. Is there any analysis of the financial impact resulting from CKD coding?**

**Our response:** We agree that this is an important and underexplored area. However, to our knowledge, there are currently no studies examining the financial impact of coded versus uncoded CKD on either patient-level or health system outcomes. This lies outside the scope of the present analysis, which was designed to evaluate mortality risks, but we recognise the value of future work to address this question.

**3. Has there been an age-specific analysis on CKD coding—particularly in the elderly population?**

**Our response:** Thank you for this question. Prior studies have explored the prevalence of undiagnosed CKD across different age groups. Our study, however, was specifically designed to evaluate the association between coding status and mortality. Age group was included in our survival models but could not be retained as a standard covariate due to violation of the proportional hazards assumption. Instead, we modelled age as a stratified variable, which permitted separate baseline hazard functions for each age group while preserving the validity of the proportional hazards assumption.

**Reviewer #2:**

Thank you for giving me the opportunity to review the manuscript. This is a retrospective cohort study of patients with stage 3–5 chronic kidney disease (CKD). The authors reported that uncoded CKD was associated with a higher risk of all-cause and coronavirus disease 2019 (COVID-19) mortality. Although this is a thought-provoking study, several concerns need to be addressed.

**Major comments**
**1. My greatest concern is statistical analysis. The aim of this study was to investigate the association between coding of CKD and all-cause and COVID-19 mortality among patients with stage 3–5 CKD. Therefore, I would suggest that the authors include all patients with stage 3–5 CKD in the main analysis. After conducting this main analysis, the authors need to perform the same analysis by subgroups of CKD (stages 3 and 4 CKD).**

**Our response:** We thank the reviewer for this important comment. While the overall aim of our study was to examine the association between coding status and both all-cause and COVID-19 mortality in patients with CKD stages 3–5, we felt that presenting a single risk estimate across a heterogeneous aggregated group of patients with stages 3-5 CKD would be less methodologically sound or clinically meaningful. The reason for this is that mortality risk, coding prevalence, and management strategies differ markedly by CKD stage; for example, fewer than 2% of patients with stage 5 CKD were uncoded in our dataset compared with 20% for stage 3 CKD. Additionally, conducting survival analyses for CKD stage 5 where there were only 6 uncoded patients not only violated proportional hazards assumptions but also produced unstable and unreliable parameter estimates.

More importantly, from a research and clinical perspective, knowing whether coding is associated with increased mortality for such a large and heterogeneous group provides little actionable insight. The benchmark for this type of work was set by the National CKD Audit, which demonstrated that clinically meaningful estimates of mortality risk emerge when stages are analysed separately rather than in aggregate. In keeping with this precedent, we therefore restricted inferential analyses to stages 3 and 4, where sample sizes were sufficient and model assumptions were met, and presented descriptive analyses for stage 5.

As such we have clarified the aims of the study in the methods section: *“The aims of this research, codesigned with a CKD patient involvement group, were to quantify the impact of clinical coding on risk of death and COVID-19 death in patients with CKD stages 3-5,* ***by CKD stage****, in primary care in Greater Manchester, England.”*

We have also clarified this rationale in out statistical plan within the methods section: *“Survival analyses were conducted by CKD stage to allow for coding status to be compared within each group, in line with existing research in this area^3,10,19^.”*

**2. Given that the aim of this study was to investigate the association between coding of CKD and mortality, I would suggest that the authors compare the two groups in Table 1: patients with coded CKD and those with uncoded CKD.**

**Our response:** We have retained Table 1 in the manuscript to preserve clarity, as stratifying by coding status risked making the table unwieldy. However, in line with the reviewer’s suggestion we have created an updated version of Table 1 that stratifies CKD stages by coding status; this new version is now included as S2. We note that there were only 6 patients with uncoded CKD stage 5. As the governance procedures for use of the GMCR dataset require suppression of low cell counts (<5), reporting covariate prevalence in this group would have necessitated suppressing all values, rendering the table uninformative. Accordingly, in S2, we present covariate prevalence for CKD stage 5 patients in aggregate (coded and uncoded combined).

**3. Considering the study design and limitations of this study, I would suggest that the authors tone down the Conclusion section.**

**Our response:** Please see our updated discussion and conclusions section. Specially in the discussion we have removed a few paragraphs whilst rewriting a main section to balance the conclusions whilst incorporating your suggestions for additional evidence, as follows: *“Uncoded CKD is common[3,9,10] and barriers to coding CKD have been described[4]. The prevalence of undiagnosed CKD is higher in other high-income countries (stage 3 prevalence range: 61.6%-95.5%)[22] than in our analyses (stage 3 uncoded prevalence: 18.9%) in part because we excluded uncoded patients at baseline that were coded during the study period. In the UK, strategic initiatives to improve CKD coding practices in primary care[4,23,24] alongside collaborations between primary care and nephrologists may also contribute to lower rates of uncoded CKD in our primary care population[20,23,25–27]. These collaborations in England are supported by national auditing tools (CVD PREVENT[11]) which allows clinicians to quickly estimate the number of patients at practice and regional level with uncoded CKD. Evidence from Japan, underscores the benefits of primary care-nephrology collaborations for managing patients with CKD stage 5 – collaborative practices were associated with a lower hazard of infection-related hospitalisation (HR 0.36; CIs 0.15-0.87)[28].*

*Despite these efforts, uncoded CKD remains a significant challenge. A recent study in England showed uncoded CKD prevalence at hospital discharge was 58.7% with only 6.7% receiving a diagnostic CKD code during hospitalisation[20]. This variability across settings illustrates the heterogeneity in coding practices and the universality of the problem requiring a collaborative population health approach[20].”*

We have also rewritten the conclusion as follows: *“Clinical coding is an intervention associated with a reduced hazard of death and a possibly reduced hazard of COVID-19 death for patients with CKD stages 3 and 4 emphasising the importance of coding not only in clinical record keeping but also its potential in improving health outcomes.****”***

**4. I would suggest that the authors clearly describe the definition of confirmed SARS-CoV-2 infection. Were the patients diagnosed with COVID-19 based on positive PCR or antigen test results?**

**Our response:** We have clarified in the Methods section (page 5) that *“Primary outcomes included all-cause mortality and COVID-19 death (within 28 days of a positive COVID-19 test within a patient’s EHR).”*

**5) I would suggest adding discussions regarding the high proportion of coded CKD and the role of primary care physicians in England. Surprisingly, 81.1% of the patients were coded as having CKD in this study. A previous study using databases from five countries (France, Germany, Italy, Japan, and the United States) reported consistently high proportions of patients with undiagnosed stage 3 CKD [Ref #1].** **This discrepancy can, at least in part, be attributed to the quality of primary care physicians in England. A previous study reported that preventing hospitalization is a key role for primary care physicians, even during the COVID-19 pandemic [Ref #2]. Primary care physicians play an important role in the field of nephrology. A cohort study in Canada reported that patients with stage 1–4 CKD treated by primary care physicians had a lower risk of hospitalization for heart failure and hyperkalemia than those who were not treated by primary care physicians [Ref #3]. Regarding infections, primary care physician-nephrologist collaboration was associated with a lower risk of infection-related hospitalization among patients with stage 5 CKD [Ref #4]. The above discussions on the role of primary care physicians will enhance the importance of this study.**

- Ref #1 Tangri N, et al. Prevalence of undiagnosed stage 3 chronic kidney disease in France, Germany, Italy, Japan and the USA: results from the multinational observational REVEAL-CKD study. BMJ Open. 2023;13:e067386.
- Ref #2 Aoki T, et al. Impact of primary care attributes on hospitalization during the COVID-19 pandemic: a nationwide prospective cohort study in Japan. Ann Fam Med 2023;21:27–32.
- Ref #3 Wiebe N, et al. Potentially preventable hospitalization as a complication of CKD: a cohort study. Am J Kidney Dis 2014;64:230–238.
- Ref #4 Murakami M et al. Association between primary care physician–nephrologist collaboration and clinical outcomes in patients with stage 5 chronic kidney disease: a JOINT‑KD cohort study. J Nephrol 2025;38:1385–1394.

**Our response:** Thank you for these suggestions. Please see our updated discussion where we have included 2 of these suggested references (Tangri and Murakami) to strengthen our messaging around the benefits of collaborative primary care and nephrology approaches in managing CKD. Please see the reply to point 3 for inclusion of these data.

**6) I would suggest adding a supplementary figure showing the flowchart of the study participants because only 47,628 patients with stage 3–5 CKD out of 2.45 million patients were included in the analysis.**

**Our response:** Thank you for this suggestion. Please find a flow chart of the study participants within figure 1.

**7) I would suggest adding the causes of CKD to Table 1. It is unclear whether diseases, such as diabetes and hypertension, are the causes of CKD or comorbidities.**

**Our response:** We agree this would be nice to include. Unfortunately, this is not possible as this information is contained within free text information within the EHR which we do not have access to in the GMCR.

**Minor comments**
**8) I would suggest that the authors clarify why patients who died due to non-COVID-19 were excluded from the analysis (page 6). In the Cox proportional hazards model, the patients who experienced such events are censored. However, in the Fine–Gray model, such events are treated as competing risk events.**

**Our response:** We agree that non-COVID-19 deaths represent an important competing risk for COVID-19 mortality. In our primary Cox proportional hazards analyses, we excluded non-COVID-19 deaths to isolate the specific association between coding status and risk of COVID-19 death. To address the competing risk directly, we subsequently included non-COVID-19 deaths in Fine–Gray competing risk models as sensitivity analyses, which yielded very similar results. We have now updated our methods section to make this more explicit: *“For hazard of all-cause mortality, all patients were included.* ***For hazard of COVID-19 mortality, in order to isolate the impact of coding on risk of COVID-19 death,*** *non-COVID deaths after either a) the first suspected COVID-19 death in the UK (01/03/2020) or b) the date of first vaccine availability in the UK (8/12/2020), were excluded.”*

**9) I would suggest adding a supplementary Figure showing the flowchart of the study participants because only 47,628 patients with stage 3–5 CKD out of 2.45 million patients were included in the analysis.**

**Our response:** Thank you for this suggestion. Please find a flow chart of the study participants within figure 1.

**10) I would suggest that the authors clearly describe whether the hazard ratio is unadjusted or adjusted throughout the manuscript. The term “hazard ratio” is ambiguous for readers to understand the results of this study.**

**Our response:** Please see our updated manuscript where we reviewed all documentation of hazard ratios and clearly stated that they were adjusted.

**11) In relation to the above comment, I would suggest that Tables 2 and 3 list the adjusted hazard ratios, 95% confidence intervals, and p-values in that order.**

**Our response:** Please see our updated tables where we have reordered the columns according to your suggestion.

**12) I would suggest that the authors revise the Descriptive analyses by CKD stage section. As mentioned above, descriptions according to the CKD stage are not necessary because this is a subgroup of study participants. Moreover, these sentences appear redundant.**

**Our response:** We have revised the manuscript accordingly. The descriptive analyses for CKD stages 3 and 4 have been removed, as these groups are primarily included in the inferential analyses. However, we have retained a brief descriptive summary for CKD stage 5, as survival analyses could not be conducted for this group and descriptive data provide important context.

**13) I would suggest combining Figures 3 with 4, and 5 with 6. A Kaplan–Meier curve with an enlarged y-axis is commonly incorporated within the area of the original Kaplan–Meier curve.**

**Our response:** Due to limitations within the virtual environment used to access the dataset we could only download specific R packages. Insetting of graphs within graphs was only achievable using specific packages in R which we were unable to use in this virtual environment. However, at present, the currents graphs are still sufficient for visually communicating the information within each survival plot.

**14) I would suggest that the author suggests clearly stating the p-value (page 15). when the p-value is 0.05, please include three decimal places (e.g., 0.048).**

**Our response:** Please see our updated results section where the borderline p-value has been updated (p= 0.045).

**15) I would suggest that the authors recheck the reference list. For example, #20 of Reference needs to be revised as follows:** BMC Nephrol 2025;26:1–10. → BMC Nephrol 2025;26:39.

**Our response:** Thank you for highlighting this. We have reviewed and updated our reference list according to PLOS One standards.

**16) I would suggest that the authors correct grammatical errors throughout the manuscript.**

**Our response:** Please find our revised manuscript where we have reviewed all grammatical errors.
